# Is intensive glucose control bad for critically ill patients? A systematic review and meta-analysis

**DOI:** 10.7150/ijbs.43447

**Published:** 2020-03-12

**Authors:** Ren-qi Yao, Chao Ren, Guo-sheng Wu, Yi-bing Zhu, Zhao-fan Xia, Yong-ming Yao

**Affiliations:** 1 Trauma Research Center, Fourth Medical Center of the Chinese PLA General Hospital, Beijing 100048, People's Republic of China.; 2 Department of Burn Surgery, Changhai Hospital, the Second Military Medical University, Shanghai 200433, People's Republic of China.; 3 Department of Critical Care Medicine, Fuxing Hospital, Capital Medical University, Beijing 100038, People's Republic of China.

**Keywords:** intensive glucose control, critical illness, sepsis, meta-analysis

## Abstract

**Background:** The monitoring and management of blood glucose concentration are standard practices in critical settings as hyperglycaemia has been shown close association with poorer outcomes. Several meta-analyses have revealed that intensive glucose control has no benefit in decreasing short-term mortality among critically ill patients, while the studies these meta-analyses have incorporated have been largely divergent. We aim to perform a more comprehensive meta-analysis addressing this problem to provide stronger evidence.

**Methods**: We conducted comprehensive searches for relevant randomized controlled studies in online databases, including the Cochrane Library, EMBASE, and PubMed databases, up to September 1, 2018. The clinical data, which included all-cause mortality, severe hypoglycemia, need for RRT, infection resulting in sepsis, ICU mortality, 90-day mortality, 180-day mortality, and hospital and ICU lengths of stay, were screened and analyzed after data extraction. We applied odds ratios (ORs) to analyze dichotomous outcomes and mean differences for continuous outcomes with a random effects model.

**Results:** A total of 57 RCTs involving a total of 21840 patients were finally included. Patients admitted to the ICU who underwent intensive glucose control showed significantly reduced all-cause mortality (OR: 0.89; 95% CI: 0.80-1.00; *P*=0.04; I^2^=32%), reduced infection rate (OR: 0.65, 95% CI: 0.51-0.82, P=0.0002; I^2^=47%), a lower occurrence of acquired sepsis (OR: 0.80, 95% CI: 0.65-0.99, P=0.04; I^2^=0%) and shortened length of ICU stay (MD: -0.70, 95% CI: -1.21--0.19, P=0.007, I^2^=70%) when compared to the same parameters as those treated with the usual care strategy. However, patients in the intensive glucose control group presented with a significantly higher risk of severe hypoglycemia (OR: 5.63, 95% CI: 4.02-7.87, *P*<0.00001; I^2^=67%).

**Conclusions:** Critically ill patients undergoing intensive glucose control showed significantly reduced all-cause mortality, length of ICU stay and incidence of acquired infection and sepsis compared to the same parameters in patients treated with the usual care strategy, while the intensive glucose control strategy was associated with higher occurrence of severe hypoglycemic events.

## Background

For decades, hyperglycemia has been a frequent yet intractable issue in patients who are admitted to the intensive care unit (ICU), and hyperglycemia is associated with severe adverse reactions including high susceptibility to infectious complications, oxidative stress, immune dysfunction and increased mortality^1^-^7^. Accordingly, this issue prompted the management of blood glucose levels to become a crucial prevention and intervention means for critically ill patients^8^. Aiming for normoglycemia (blood glucose concentration of 80-110 mg/dL), also known as intensive glucose control, was demonstrated to be beneficial for the outcomes of patients admitted to the surgical ICU, as reported by a landmark single-center randomized controlled trial (RCT) conducted by Greet Van den Berghe and her colleagues in 2001^9^, ^10^. Subsequently, intensive blood glucose control was brought to the forefront and verified in several large RCTs among a variety of ICU categories 11-14. Nevertheless, these RCTs all failed to replicate the mortality benefit of underlying intensive blood glucose control for patients in the ICU, and the largest “Normoglycemia in Intensive Care Evaluation and Survival Using Glucose Algorithm Regulation” (NICE-SUGAR) trial even reported its potential detrimental effects 12.

All RCTs consistently concluded that intensive glucose control gave rise to an increased occurrence of hypoglycemia. Observational and prospective studies suggested that moderate and severe hypoglycemia (concentrations of blood glucose <70 mg/dL and 40mg/dL, respectively) may be independently associated with increased mortality^15^-^18^. Although there is a lack of direct and strong evidence demonstrating that hypoglycemia deteriorates the patient's prognosis, hypoglycemia might cause long-term impairment of neurocognition, which was also difficult to evaluate^19^-^22^.

The “Standards of Medical Care in Diabetes” recently developed by the American Diabetes Association (ADA) recommend the range of 140-180 mg/dL as a target level of blood glucose for the majority of critically ill patients^23^. In addition, the recommendation from the Surviving Sepsis Campaign showed that blood glucose level ≤180 mg/dL should be targeted in the management of blood glucose^24^. Clearly, the international consensus shows that intensive glucose control is not recommended for patients admitted to the ICU. However, high-quality evidence is far from adequate. In recent years, many well-designed systematic reviews and meta-analyses have been published and noted around this issue^25^-^30^, including two network meta-analyses^28^, ^29^. The divergence of the included studies may be due to a disparate search strategy and inclusion criteria. Additionally, the Grading of Recommendations Assessment, Development and Evaluation (GRADE), which is used to evaluate the quality of evidence, was not used in many previous meta-analyses.

Consequently, our objective is to perform a comprehensive and updated systematic review and meta-analysis to explore the risks and benefits of intensive glucose control compared with usual care for patients in the ICU. We also aim to provide strong evidence of the optimal blood glucose targets for critically ill patients. Moreover, sepsis remains a serious issue in the ICU and constantly leads to poor clinical outcomes and death^31^. Due to the unique pathophysiological features, septic patients suffered more often from hyperglycemia^32^, ^33^. Likewise, Neurocritical care patients are a unique subset, and they are particularly sensitive to both hyperglycemia and hypoglycemia, which may induce free radical formation, oxidative injury and trigger apoptotic pathways, thereby impairing central nervous system and worsening clinical outcomes^34^, ^35^. In addition, the strategies for glucose control are reportedly different in patients with diabetes mellitus due to aberrant metabolism of blood glucose, indicating a cautious yet specific management strategy for critically ill patients with diabetes. Prospective and retrospective trials found that the beneficial effects of treating hyperglycemia may not be the same in critically ill patients with diabetes mellitus, and lowering blood glucose excessively in this population may be detrimental^18^, ^36^-^40^. Given that, we plan to conduct subgroup analyses with four subsets: different ICU admission categories (medical ICU, surgical ICU and medical-surgical mixed ICU), critically ill patients complicated with sepsis or septic shock, critically ill patients with diabetes mellitus as well as neurocritical care patients.

## Methods

### Search strategy

We identified all studies relevant to our research by systematically searching MEDLINE, EMBASE and the Cochrane Central Register of Controlled Trials. We conceived a strategy that comprised the following combination of exploded medical subject heading (MeSH) terms and text words: "blood glucose", "insulin", “glycemic control”, “intensive care unit”, “critical care”, “critical illness”, "postoperative care", "sepsis", “myocardial infarction”, “cardiovascular surgical procedures”, “stroke”, "wounds and injury", and “septic shock”. Additionally, we used highly specific search filters furnished by the Health Information Research Unit (HIRU) at McMaster University. We performed similar searches using the EMBASE and CENTRAL databases to comprehensively incorporate all related RCTs that compared intensive versus conventional glucose control in the clinical settings of critically ill patients. Additionally, we manually screened abstracts from conferences and valid data from other published systematic reviews and meta-analyses. Our search was conducted up to September 1, 2018, and included RCTs without any language limitations.

### Study selection

#### Inclusion criteria

We incorporated studies in accordance with the following criteria: (1) all recruited patients were adults (age>18 years) with hyperglycemia; (2) all studies were incorporated that reported patients with critical illnesses (e.g., ICU); (3) the enrolled studies compared a minimum of two arms of intensive glucose control and regular glucose control in which variability in the glucose goal could be established; (4) the intervention of glucose control was limited to insulin infusion.

#### Exclusion criteria

1. Unpublished trials were excluded; 2. We excluded studies in which glucose control was not implemented by means of insulin administration or glucose-insulin-potassium infusions (GKIs); and 3. Trials were excluded if we could not obtain sufficient information on the results and intervention methods from the authors.

Two authors (RQY and CR) screened the titles and abstracts of studies independently. In the case of the potentially eligible trials without sufficiently validated details, the full-text was required for further consideration. Disagreements between the two reviewers were addressed by discussion. If a consensus could not be reached, the corresponding authors (ZFX and YMY) dealt with the disagreements.

### Data extraction and quality evaluation

Two reviewers independently accessed and extracted data from all included RCTs. First author, the number of participants, year of publication, patient baseline characteristics, setting, clinical outcomes and target glucose concentration of each trial were abstracted and recorded by using a predesigned, standardized form. In addition, severe hypoglycemic episodes referring to the status when blood glucose concentrations fell below 40 mg/dL were also recorded and analyzed. Missing data were obtained by means of contacting authors directly.

We performed assessments of risk of bias by using the Cochrane Collaboration tool. The randomization sequence, allocation concealment, blinding of personnel and participants, risk of incomplete outcome data, selective reporting bias and other sources of bias were evaluated by two authors. A score of 'low', 'high' or 'unclear' bias was attributed to each option, and then each trial was rated as having a low, moderate or high risk of bias accordingly.

The quality of evidence was assessed in line with the GRADE tools, which were processed with GRADE Pro software 3.6 (McMaster University 2014, Hamilton, Canada).

### Outcome measurements

#### Primary outcomes

We chose all-cause mortality as the primary outcome because it was the most valid criterion for the detection of which protocol of glucose control was superior. In accordance with Wiener's meta-analysis, we preferentially used data of mortality occurring during the hospital stay or within 30 days following admission^27^.

#### Secondary outcomes

The secondary outcomes included severe hypoglycemia, need for renal replacement therapy (RRT), infection resulting in sepsis, ICU mortality, 90-day mortality, 180-day mortality, hospital and ICU length of stay. The universal definition of severe hypoglycemia was a blood glucose level below 40 mg/dL. Hypoglycemia was a commonly complicated yet extremely dangerous outcome which might cause irreversible brain injury, especially for patients who underwent insulin infusion. Although precise criteria were not provided by the included RCTs, the proportion of patients who needed RRT still represented a crucial endpoint for sepsis or septic shock patients during insulin therapy. Infection and sepsis were also selected because of their predictive value of prognosis. In addition, infection rates were presented with contradictions among different trials. The incidence of sepsis was in accordance in the RCTs.

### Subgroup analyses

#### ICU setting

Given that the prognosis of critical patients may differ from disparate ICU admission, we stratified RCTs into three tiers: medical ICU (containing neurologic patients and general medical patients), surgical ICU (including patients who had undergone cardiac surgery, neurocritical surgery, abdominal surgery and general surgery) and mixed ICU (medical-surgical ICU).

#### Septic patients

Insulin infusion in various clinical settings may be different due to its particular pathophysiologic characteristics. Therefore, we performed subgroup analyses by categorizing trials into septic ICU settings to address the discrepancy of insulin therapy among patients diagnosed with sepsis or septic shock.

#### Neurocritical care patients

Hyperglycemia is detrimental to central nervous system in many dimensions, which reportedly worsen the prognosis of neurocritical care patients^19^, ^34^. Given that, we aimed to perform a subgroup analysis and compared patients-centered outcomes between two strategies regarding to patients with associated conditions, including traumatic brain injury (TBI), aneurysmal subarachnoid hemorrhage (SAH), spontaneous intracerebral hemorrhage (ICH) and ischemic stroke.

#### Diabetic patients

Unlike previously reported systematic reviews and meta-analyses in which subgroup analyses were conducted based on specific target concentrations of blood glucose under intensive control, we planned to identify whether preexisting chronic hyperglycemia may affect the goals of different glucose control. Therefore, we conducted a subgroup analysis that enrolled patients with either prediagnosed type 1 or type 2 diabetes mellitus and further compared mortality between the two arms.

### Data synthesis and statistical analysis

Our study applied ReviewManager (RevMan 5.3, Copenhagen: The Nordic Cochrane Centre, The Cochrane Collaboration, 2014) for data processing. Odds ratios (ORs) were used for analyzing dichotomous outcomes, while continuous outcomes were analyzed by mean differences. We calculated a pooled-data estimate and 95% confidence intervals (CIs) for each outcome (p value less than 0.05 indicated statistical significance). The significance of heterogeneity was evaluated by Cochran's I^2^ test provided by the software, in which I^2^>25% revealed a moderate or high degree of heterogeneity, and I^2^<25% revealed a low degree of heterogeneity; the Q test was used as well (a value below 0.10 was deemed as statistically significant). In the present work, we applied a random-effects model based on the Cochrane Collaboration recommendation in the case of meaningful heterogeneity. If no moderate or high degree of heterogeneity was present in our meta-analysis, we applied a fixed-effects model accordingly. In addition, we performed sensitivity analysis by excluding the enrolled studies one at a time from the pooled data.

We assessed the publication bias of the primary outcome by means of visually inspecting the funnel plot and conducting Begg's and Egger's tests (p value < 0.05 considered statistically significant). If publication bias existed, the trim-and-fill computation method was applied to evaluate the impact of publication bias on the interpretation of results.

## Results

### Literature search and study characteristics

**Figure 1:**
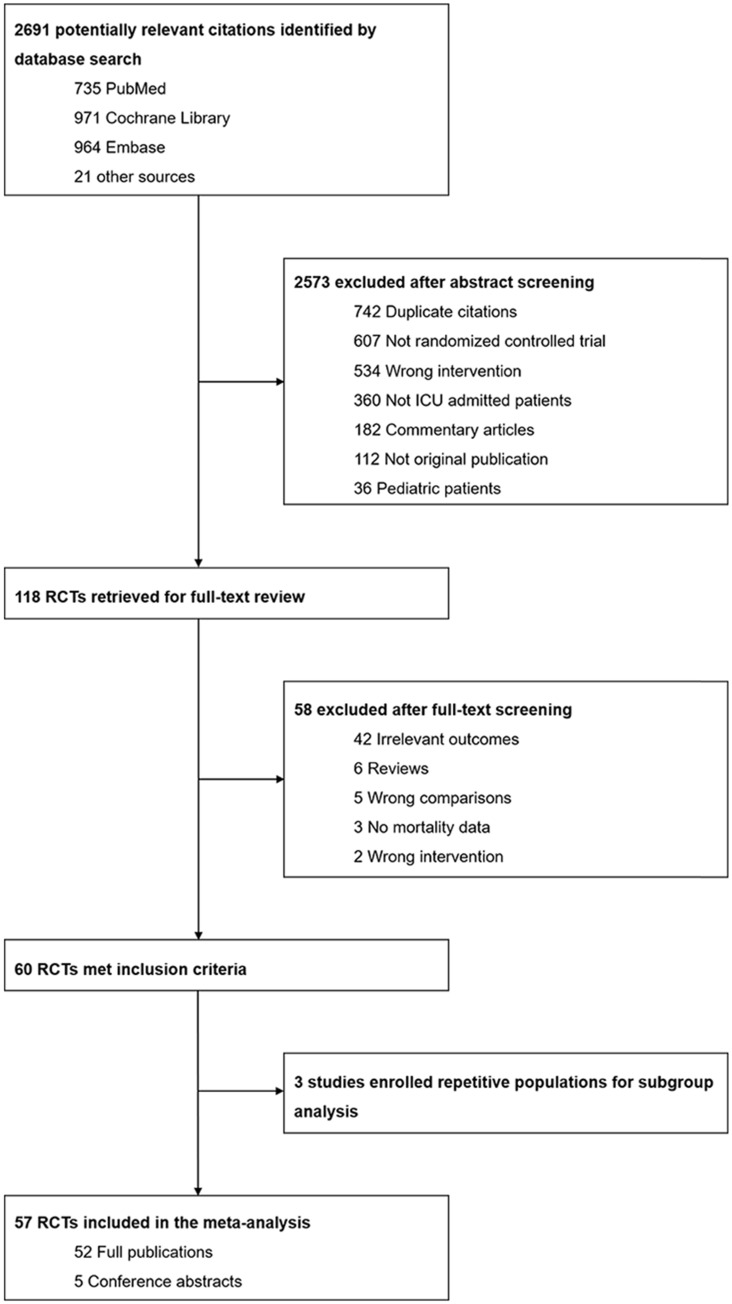
Flow chart for study selection.

We identified a total of 2691 citations through the database search (735 from MEDLINE, 971 from CENTRAL and 964 from EMBASE) and other sources (8 from conference abstracts and 13 from previously published systematic reviews and meta-analyses). After screening abstracts and titles, we removed the majority of trials on account of not being RCTs, not being conducted in an-ICU setting, not being original publications and performing the wrong comparison. Subsequently, the full texts of 118 potentially eligible RCTs were scanned. We excluded 42 trials due to them reporting irrelevant outcomes rather than the predefined primary or secondary outcomes. The detailed screening procedure is provided in **Figure 1**. Eventually, 57 trials involving a total of 21840 patients were incorporated into our systematic review and meta-analysis, which comprised 52 full publications and 5 conference abstracts^9^, ^11^-^14^, ^41^-^92^.

The characteristics of all enrolled studies are shown in **Table 1**. Trials with publication years ranging from 1991 to 2018 were implemented in diverse countries and medical centers, in which the majority of RCTs were conducted in single-center settings. Sample sizes also varied. The smallest trial enrolled only 10 patients, while the largest trial enrolled 6104 participants^12^, ^48^. Among all included studies, 29 included fewer than 100 participants, while 7 trials enrolled more than 1000 patients. The protocols of intensive insulin therapy were diverse: blood glucose concentrations less than 99 mg/dL contributed to the strictest target of glucose control, while the moderate glucose goal was between 120 mg/dL and 160 mg/dL and was applied by Giakoumidakis and his colleagues^61^. Most of the trials implemented tight glucose control with a glucose goal within the range of 80 mg/dL to 120 mg/dL. This systematic review and meta-analysis covered a large number of clinical ICU settings. Sixteen trials were conducted in the medical ICU, while 18 trials recruited only surgical patients. The remaining RCTs were conducted in mixed (surgical -medical) ICU settings. Moreover, 13 trials enrolled patients diagnosed with sepsis or septic shock, and of these trials, 10 RCTs enrolled exclusively septic patients^11^, ^12^, ^42^, ^43^, ^51^, ^58^, ^67^, ^69^, ^76^, ^81^, ^83^, ^91^, ^92^. The baseline characteristics of all participants were the same between the two arms (intensive glucose control vs conventional glucose control). In addition, the occurrence of type I or type II diabetes mellitus ranged from 0% to 100% among the included studies.

### Primary outcome: All-cause mortality

**Figure 2:**
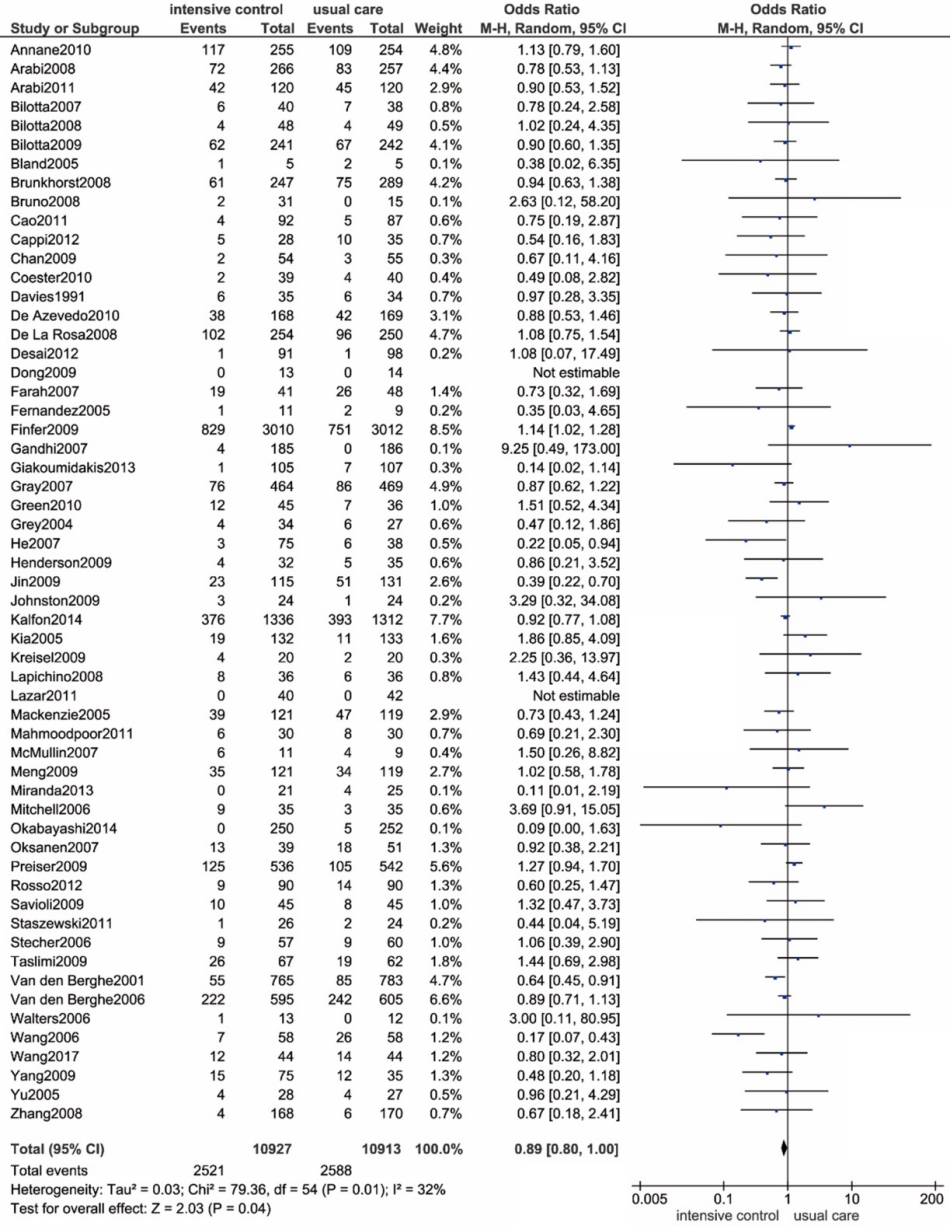
** Forest plot of all-cause mortality comparing intensive glucose control to usual care in ICU patients. CI confidence interval.** M-H: Mantel-Haenszel.

Within this meta-analysis, 36 trials reported hospital mortality directly, and 21 studies provided useable data that conformed to our pre-claimed definition. By graphing a forest plot of all eligible RCTs, we found that critical ill patients undergoing intensive glucose control presented with a reduced risk of all-cause death compared to those undergoing usual care, and the difference was statistically significant (OR: 0.89; 95% CI: 0.80-1.00; *P*=0.04; I^2^=32%) (**Figure 2**). The test for heterogeneity indicated that the trial by Finfer S et al. was an outlier, which might have been due to its overly large sample size. The exclusion of this trial significantly diminished heterogeneity without affecting the conclusion (OR: 0.88; 95% CI: 0.79-0.98; *P*=0.02; I^2^=22%)^12^.

**Figure 3:**
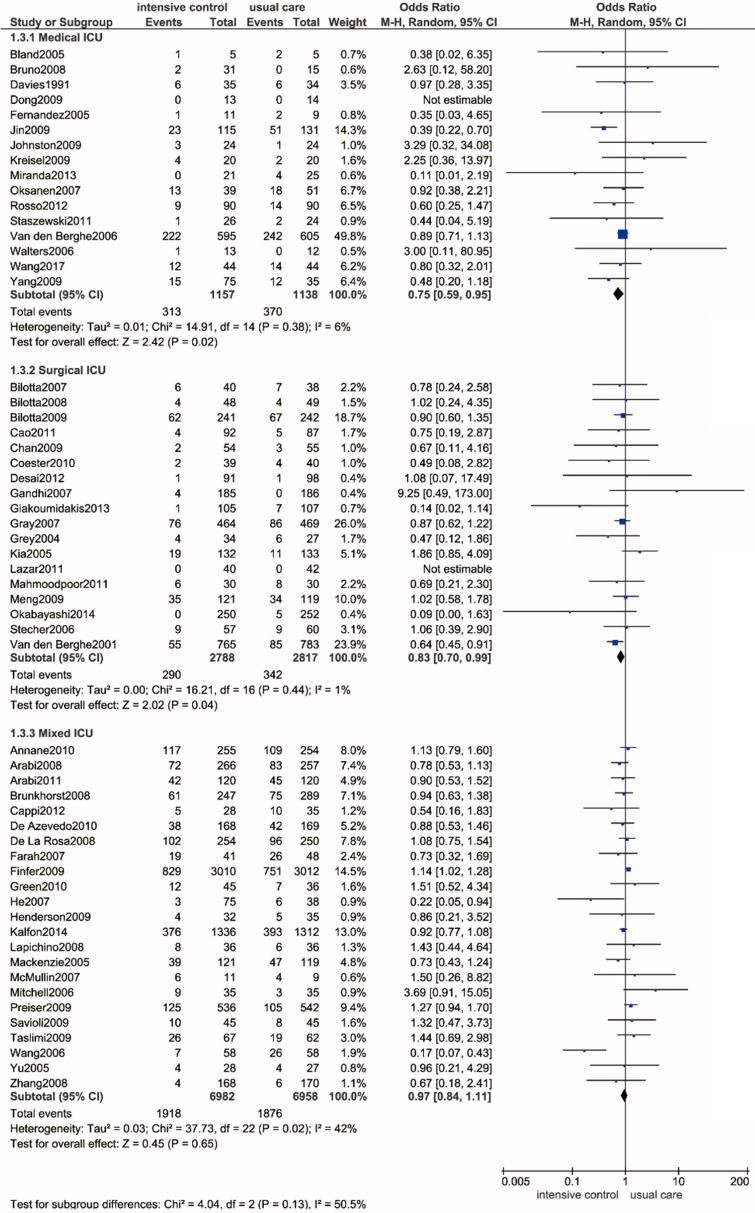
** Forest plot of all-cause mortality comparing intensive glucose control to usual care stratified by ICU setting.** ICU, intensive care unit. CI confidence interval. M-H: Mantel-Haenszel.

As shown in** Figure 3** and** Table 2**, we performed a subgroup analysis based on different ICU settings, including medical ICU, surgical ICU and mixed (medical-surgical) ICU settings. Intensive glucose control significantly reduced all-cause mortality compared with usual care in the medical ICU settings (OR: 0.75; 95% CI: 0.59-0.95; *P*=0.02; I^2^=6%) as well as the surgical ICU settings (OR: 0.83; 95% CI: 0.70-0.99; *P*=0.04; I^2^=1%). However, a contradictory result was noted in the mixed ICU settings, as evidenced by no significant difference in all-cause mortality between intensive control and conventional strategy (OR: 0.97; 95% CI: 0.84-1.11; *P*=0.65; I^2^=42%). The heterogeneity test solely identified a moderate degree of heterogeneity among patients in a mixed ICU setting, with an I^2^ value of 42%. Sensitivity analysis revealed the trial by Wang et al. as the main source of heterogeneity^87^. When the outlying study was removed, heterogeneity was then diminished significantly (I^2^=11%). We assumed this heterogeneity might be mainly due to the discrepancy in baseline characteristics between the two arms.

### Secondary outcome: mortality in different phases, severe hypoglycemia, need for RRT, infection and sepsis, length of hospital stay and length of ICU stay

Pooled effect and characteristic of each secondary endpoint were summarized in **Table 3**. The 90-day and 180-day mortality rates were reported in 11 trials and 9 trials, respectively, and ICU mortality was analyzed in 16 trails^9^, ^11^-^14^, ^42^-^47^, ^49^, ^53^, ^55^, ^56^, ^59^, ^63^, ^67^, ^70^, ^71^, ^73^-^75^, ^77^, ^80^, ^82^, ^85^, ^88^, ^89^, ^91^. No significant difference was observed in 90-day mortality (OR: 1.04, 95% CI: 0.95-1.13, *P*=0.39; I^2^=3%), 180-day mortality (OR: 0.99, 95% CI: 0.81-1.20, *P*=0.89; I^2^=0%) and ICU mortality (OR: 0.95, 95% CI: 0.85-1.06, *P*=0.36; I^2^=19%) between critically ill patients undergoing intensive glucose control and those receiving usual care (**Supplemental Figure S1-S3**).

**Figure 4:**
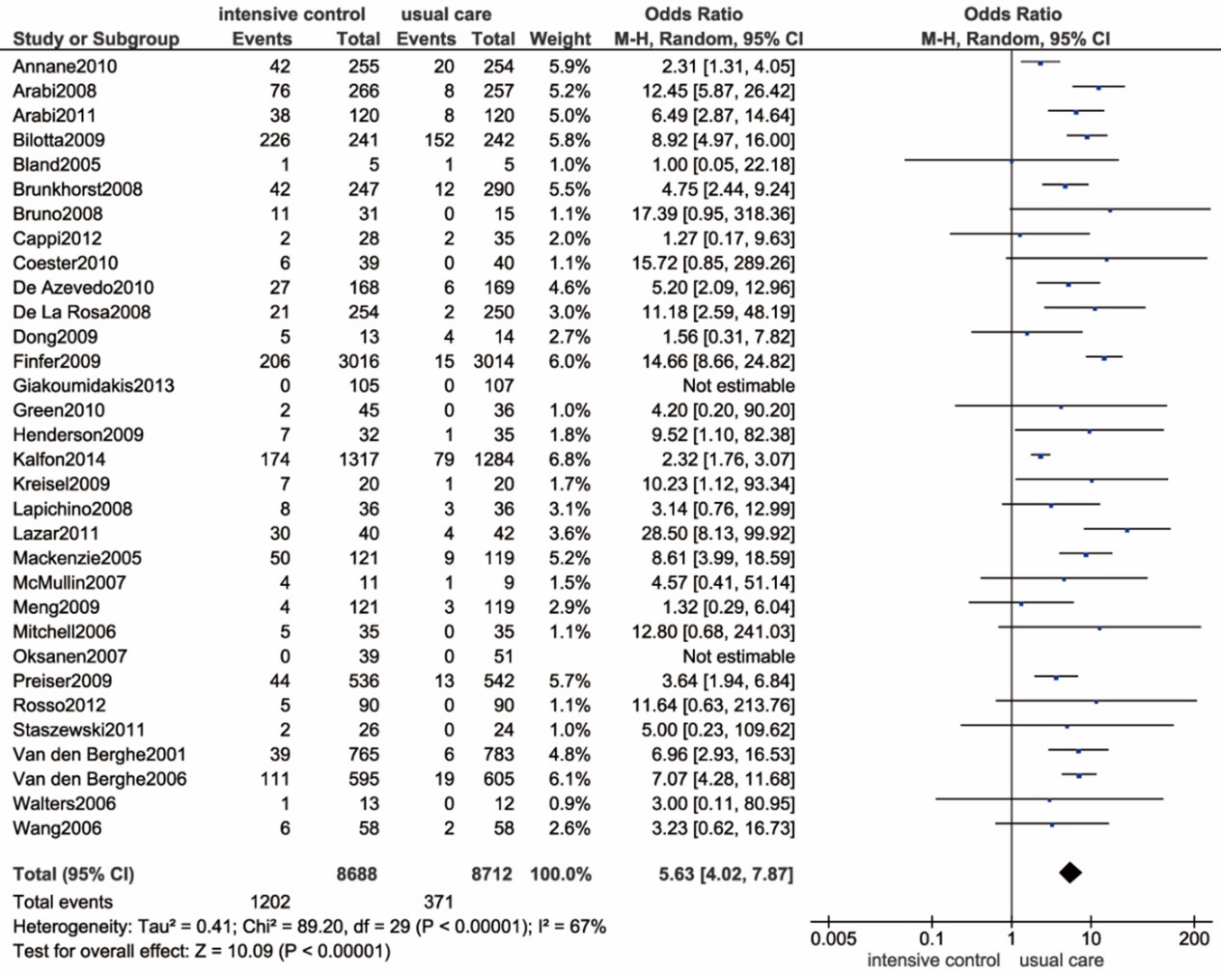
** Forest plot of severe hypoglycemia comparing intensive glucose control to usual care in ICU patients.** CI confidence interval. M-H: Mantel-Haenszel.

Severe hypoglycemia episodes were reported in 32 trials 9, 11-14, 42-44, 46, 48, 49, 51, 53, 55, 56, 58, 61, 63, 66-68, 71, 72, 75, 77, 79, 80, 82, 84, 86, 87, 89. Patients in 2 trials did not develop severe hypoglycemia in any arms^61^, ^79^. The pooled data are presented in** Figure 4**, which revealed a significantly increased risk of severe hypoglycemia in the intensive glucose control group (OR: 5.63, 95% CI: 4.02-7.87 *P*<0.00001; I^2^=67%). To address the high degree of heterogeneity, we identified a study implemented by Kalfon et al. as the major source of heterogeneity by conducting a sensitivity analysis^13^. In fact, the I^2^ was reduced to 47% after removing this trial. Additionally, we performed a subgroup analysis by stratifying ICU settings, which consistently resulted in an increased risk of hypoglycemia (**Supplemental Figure S4**). Trials that were conducted in medical ICU did not reveal evident heterogeneity, while both the surgical and mixed ICU subgroup showed a high degree of heterogeneity (I^2^=59%, I^2^=75%). By performing a meta-regression analysis, we found no association between the incidence of severe hypoglycemia and several potential variables, including publication year (*P*=0.289), patient admission category (*P*=0.116 and* P*=0.637), age (*P*=0.942), proportion of diabetic patients (*P*=0.088), and sample size (*P*=0.520) (**Supplemental Figure S5**). Likewise, we could not identify the outlying study in the sensitivity analysis.

The proportion of patients who needed RRT was reported in 16 trials^11^, ^12^, ^43^, ^44^, ^51^-^53^, ^55^-^57^, ^59^, ^60^, ^66^, ^74^, ^75^, ^91^. The pooled data did not show an increased risk of RRT caused by intensive glucose control (OR: 1.07, 95% CI: 0.95-1.20, *P*=0.29; I^2^=0%) (**Supplemental Figure S6**).

Rates of infection were documented in 19 trials^13^, ^42^, ^43^, ^45^-^47^, ^50^, ^52^, ^55^-^57^, ^59^, ^63^, ^65^, ^66^, ^75^, ^88^, ^89^, ^92^. As presented in **Supplemental Figure S7,** we found a significantly decreased risk of infection associated with employing intensive glucose control when compared with usual care (OR: 0.65, 95% CI: 0.51-0.82, *P*=0.0002; I^2^=47%). This finding was the same for the acquired sepsis rate, as reported by 12 trials^9^, ^43^-^46^, ^50^, ^52^, ^53^, ^59^, ^74^, ^88^, ^89^. The intensive control protocol was correlated with a significant reduction in acquired sepsis (OR: 0.80, 95% CI: 0.65-0.99, *P*=0.04; I^2^=0%) (**Supplemental Figure S8**).

A total of 27 trials reported evidence of length of ICU stay^9^, ^11^, ^12^, ^42^-^44^, ^46^, ^52^, ^53^, ^55^, ^56^, ^59^-^61^, ^63^, ^65^-^67^, ^69^, ^72^, ^75^, ^80^, ^85^, ^87^-^89^, ^92^. It should be noted that 16 trials provided the data as median and interquartile range^9^, ^11^, ^12^, ^42^, ^46^, ^55^, ^56^, ^63^, ^65^-^67^, ^75^, ^80^, ^89^, ^92^. By pooling data from the remaining 12 trials, we demonstrated that intensive glucose control significantly shortened the length of ICU stay in comparison with usual care (MD: -0.70, 95% CI: -1.21--0.19, *P*=0.007, I^2^=70%)^43^, ^44^, ^52^, ^53^, ^59^-^61^, ^69^, ^72^, ^85^, ^87^, ^88^ (**Supplemental Figure S9**). We stratified all included trials by ICU setting and conducted a subgroup analysis. Heterogeneity was resolved in each subset of trials. (**Supplemental Figure S10**). Consistent results were detected in both the medical ICU and mixed ICU settings, while no significant association was identified in the length of ICU stay between intensive glucose control and usual care groups in the surgical ICU setting.

Length of hospital stay was documented in 15 trials, but only 8 studies were eligible for analysis^12^, ^42^-^44^, ^50^, ^52^, ^60^, ^61^, ^63^, ^66^, ^72^, ^75^, ^78^, ^80^, ^84^. A pooled-estimate revealed that reduction in the length of hospital stay was associated with intensive glucose control when compared with usual care (MD: -1.29, 95% CI: -2.56--0.01, *P*=0.05, I^2^=61%)^43^, ^44^, ^52^, ^60^, ^61^, ^72^, ^78^, ^84^ (**Supplemental Figure S11**). By performing sensitivity analysis, we found that the trial conducted by Okabayashi and his colleagues was the main source of heterogeneity^78^. The exclusion of outliers significantly lowered the heterogeneity (I^2^=41%) while simultaneously changing the finding to a nonsignificant reduction in the length of hospital stay (*P*=0.16). Therefore, intensive glucose control showed no impact on the length of hospital stay based on current evidences.

### Subgroup analysis

#### ICU settings

Stratifying trials according to different ICU settings was conducted for several outcomes, including all-cause mortality, severe hypoglycemia and length of ICU stay. The purpose of this subgroup analysis was to verify the consistency of major findings among distinct populations and simultaneously address a high degree of heterogeneity. The detailed information of each subgroup analysis was presented in **Table 2**.

#### Septic patients

**Figure 5:**
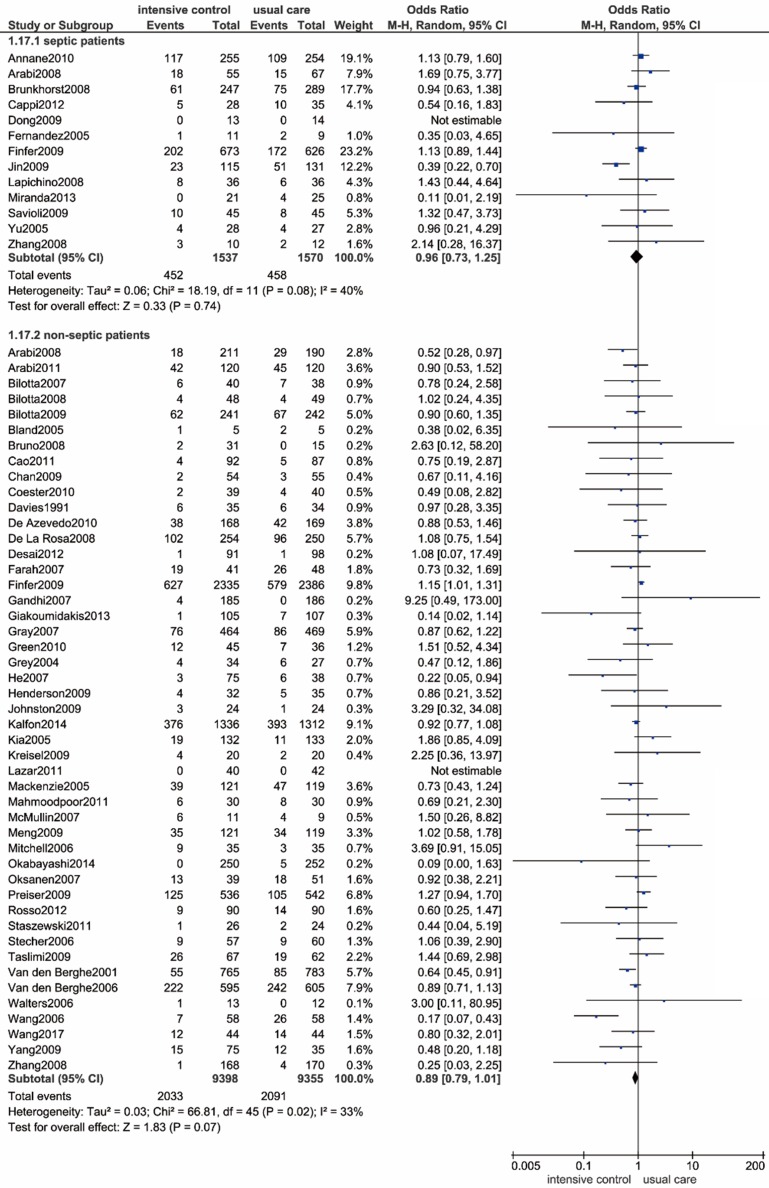
** Forest plot of all-cause mortality comparing intensive glucose control to usual care in patients with or without sepsis or septic shock.** CI confidence interval. M-H: Mantel-Haenszel.

We performed a subgroup analysis in patients diagnosed with sepsis or septic shock, and a total of 13 trials containing 3107 eligible patients were enrolled accordingly^11^, ^12^, ^42^, ^43^, ^51^, ^58^, ^67^, ^69^, ^76^, ^81^, ^83^, ^91^, ^92^. As shown in** Figure 5**, we found no significant relationship between all-cause mortality and the intensive glucose control strategy (OR: 0.96, 95% CI: 0.73-1.25, *P*=0.74; I^2^=40%). The exclusion of the outlying trial conducted by Jin et al. radically eliminated heterogeneity (I^2^=0%), and the outcome remained unchanged^69^.

#### Neurocritical care patients

**Figure 6:**
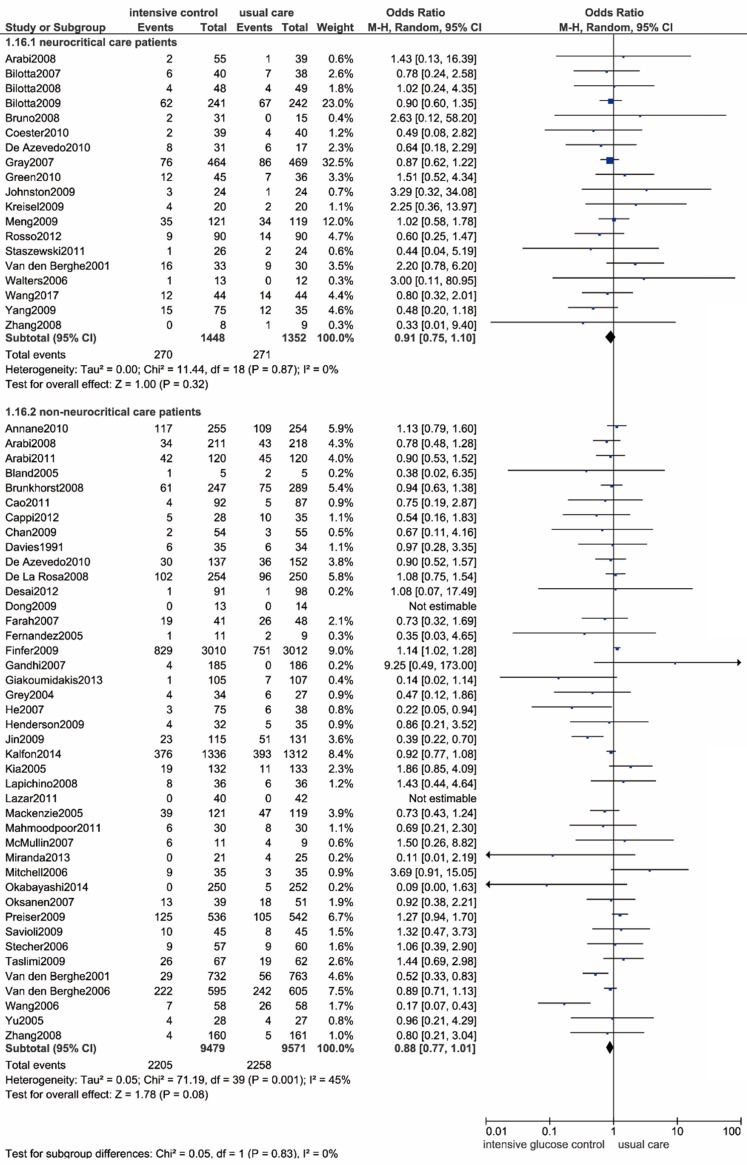
** Forest plot of all-cause mortality comparing intensive glucose control to usual care in neurocritical care or non-neurocritical care patients.** CI confidence interval. M-H: Mantel-Haenszel.

By enrolling 19 trials with 2800 neurocritical care patients^9^, ^43^, ^45^-^47^, ^49^, ^53^, ^55^, ^62^, ^63^, ^70^, ^71^, ^82^, ^84^, ^86^, ^88^-^90^, ^92^, we haven't observed statistically significance in all-cause mortality between intensive glucose control strategy and usual care (OR: 0.91, 95% CI: 0.75-1.10, *P*=0.32; I^2^=0%) (**Figure 6**).

#### Diabetic patients

**Figure 7:**
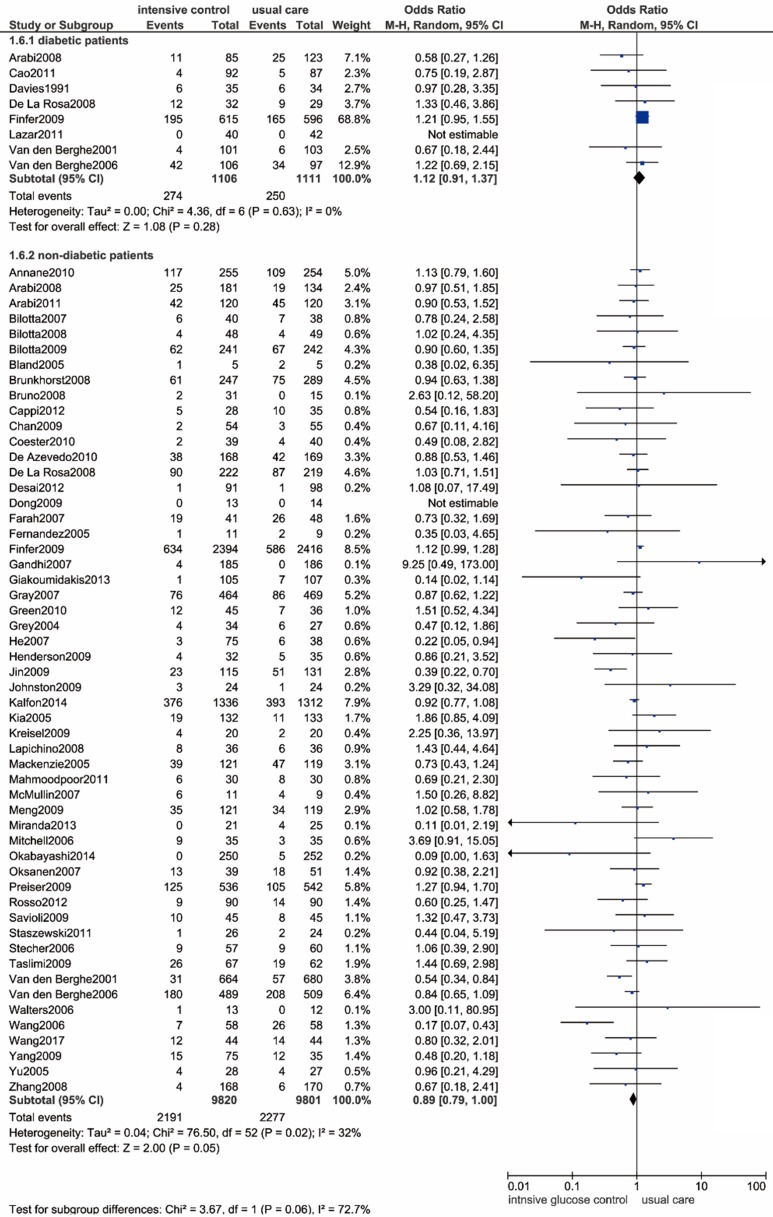
** Forest plot of all-cause mortality comparing intensive glucose control to usual care in patients who have previously been diagnosed with or without diabetes mellitus.** CI confidence interval. M-H: Mantel-Haenszel.

We included 8 studies that included 2217 patients with previously diagnosed diabetes 9, 12, 14, 43,50,54,56,72. There were no significant differences in all-cause mortality between the two glucose control methods (OR: 1.12, 95% CI: 0.91-1.37, *P*=0.28, I^2^=0%) (**Figure 7**).

### Quality of evidence and risk of bias

We listed the summary of findings for the outcomes of interest and the levels of evidence in **Table 3** and** Supplemental Table S1**. The primary endpoint was ranked as outcome with low quality of evidence due to inconsistency across enrolled studies and potential publication bias. The qualities of majority of secondary outcome data, including mortality associated with different phases, severe hypoglycemia infection and sepsis were all ranked as moderate. However, RRT as well as the lengths of hospital and ICU stays displayed low quality.

Most of the RCTs met the randomization requirements and used rational distribution methods. In each of the included trials, it was particularly challenging to blind the attending physicians and nurses to the outcome assessment based on the nature of the intervention, which inevitably resulted in a high risk of performance bias. Five trials that were reported in conference abstracts had high percentages of unclear risks (**Supplemental Figure S12**).

### Publication bias

We constructed a funnel plot to assess the possible publication bias of all-cause mortality and severe hypoglycemia (**Supplemental Figure S13-S14**). By visually inspecting the funnel plot, we found no evidence of publication bias for severe hypoglycemia but an evident asymmetry for the primary outcome of all-cause mortality. Furthermore, we used Begg's test and Egger's test to evaluate the funnel plots of both outcomes, which showed no statistically significant evidence of publication bias for severe hypoglycemia (Egger's test: =0.28; Begg's test: p=0.2), while a significant publication bias was detected for all-cause mortality (Egger's test: p=0.03; Begg's test: p=0.99) (**Supplemental Figure S13-S14**). By further performing trim-and-fill computation, the pooled effect remained unchanged. Therefore, we demonstrated that the primary outcome was not impacted by the effect of publication bias.

## Discussion

In our systematic review and meta-analysis of RCTs comparing intensive glucose control with usual care in critically ill patients, we found that intensive glucose control might potentially reduce the risk of all-cause death, infection and acquired sepsis. However, the all-cause mortality benefits were limited to the medical and surgical ICU settings, while no significant difference was identified for all-cause mortality between intensive glucose control and usual care in a mixed medical-surgical ICU setting. In addition, we did not observe any beneficial effects of intensive glucose control on mortality in other phases of follow-up, including 90-day, 180-day and ICU-stay follow-ups. We also found no correlation between intensive glucose control and increased risk of RRT requirement. On the other hand, we found an approximately 6-fold increase in the occurrence of severe hypoglycemia in critically ill patients who received intensive glucose control compared with the patients who received usual care. Although there was high heterogeneity within the analysis of this outcome, the risk of severe hypoglycemia was consistently increased in association with any type of ICU admission in the intensive control arm, which indicated strong relevance. Moreover, a significant shorter length of ICU stay was observed in patients treated with intensive glucose control when compared with those treated with usual care, but the findings solely applied to the medical ICU and mixed ICU settings but not in surgical ICU setting. Likewise, we found no significant reduction in the length of hospital stay. Altogether, we found that intensive glucose control benefited critically ill patients in many dimensions. Although intensive glucose control was associated with a higher risk of hypoglycemia, we had no reason to suspect that the risk of hypoglycemia offset the benefits of intensive glucose control in reducing mortality, infection rate and duration of ICU stay.

### Relation to prior works and interpretations

Strikingly, our meta-analysis drew discrepant conclusions when compared with the conclusions of previously published meta-analyses 25-29. A landmark meta-analysis by Wiener and his colleagues concluded that tight glucose control was not associated with a significant reduction in hospital mortality in the wide spectrum of critically ill patients^27^. While the updated study by Griesdale et al suggested that intensive glucose control might benefit patients in the surgical ICU, they failed to demonstrate any mortality benefit among medical ICU patients^12^, ^25^. In addition, two recently published network meta-analyses consistently revealed no significant differences in the risk of mortality among four blood glucose ranges for critically ill patients^28^, ^29^. However, two other meta-analyses reported a reduction in the risk of acquired sepsis in the surgical ICU setting^26^, ^27^. For the first time, the present work reported a significant reduction in ICU stay in patients under intensive glucose control, which has not been previously chosen as a secondary outcome. The significantly increased risk of severe hypoglycemia in the intensive glucose control group was in line with the findings of prior meta-analyses^25^-^29^.

The first systematic review and meta-analysis that specifically concentrated on glucose management among septic patients was conducted by Song and his colleagues^30^. Our study updated Song's work by adding two additional trials 76, 81. Indeed, septic patients were more susceptible to glucose variability, which was independently associated with higher mortality rates than hyperglycemia 33, 93-96. Therefore, our finding was in line with the latest version of the guidelines, which recommended a moderate glucose goal (blood glucose concentration<180 mg/ dL)^24^.

We further performed a subgroup analysis of critically ill patients who were pre-diagnosed with diabetes, and found no significant difference between the two arms. We assumed that the analysis might need to include more RCTs, in which chronic hyperglycemia status (diabetes mellitus) should be taken into account 10, 21. Likewise, we conducted a meta-regression to determine whether the prevalence of diabetes at baseline was related to the risk of mortality and found a negative association, suggesting that a history of diabetes was not an independent risk factor for all-cause mortality.

As noted, hyperglycemia leads to several adverse effects on fluid balance, immune function and inflammation^97^, ^98^. The present work revealed a significantly increased risk of severe hypoglycemia in patients treated with intensive glucose control, which was consistent with previously published meta-analyses and several prospective, randomized controlled trials^12^, ^13^, ^25^, ^27^-^29^, ^42^. As documented previously, hypoglycemia was associated with an increased risk of mortality and might act as an independent hazard^9^, ^15^-^18^, ^42^. However, the direct relationship between hypoglycemia and worse prognosis in the short and long-term follow-up remained unclear^10^, ^20^, ^22^, as neurologic damage caused by hypoglycemia was only explored in animal models^99^-^101^.

We initially reported a significant reduction in the duration of ICU stay when patients underwent intensive glucose control. Nevertheless, the result of subgroup analysis of patients admitted to the medical ICU must be interpreted prudently because only two studies were enrolled with only 159 patients in the intensive control arm and 175 patients in the usual care arm 69, 88.

### Limitations

Although the present meta-analysis of 57 randomized controlled trials was the most comprehensive meta-analysis, several limitations were inevitable during the implementation of this study. First, we found a significant publication bias for the primary outcome by using Begg's and Egger's test. Although we further confirmed that our conclusions could not be reversed by publication bias via conducting trim-and-fill tests, the potential effect of publication bias could not be entirely ruled out due to several limitations of this method. In addition, the test of heterogeneity revealed a moderate degree of heterogeneity, which was resolved by means of sensitivity analysis. However, many factors, such as feeding regimen, glucose control methods and the use of monitoring devices, still need to be fully elucidated in regard to whether they contributed to the heterogeneity among the included studies. Second, our search strategy seemed to be loose and covered almost all relevant trials addressing the usage of intensive glucose control among critically ill patients, which have been incorporated in previously published systematic reviews and meta-analyses 25-30, 35. Given that, we screened our included RCTs cautiously and carefully considered eligibility for every enrolled trials. In addition, we took many trials with small sample sizes into account, which may influence the pooled data estimates. Third, we did not stratify the range of target blood glucose into subgroups because almost all trials presented with the same intensive glucose control protocol, in which the glucose goal was within the range of 80 mg/dL to 120 mg/dL. Besides, it should be noted that a few studies did exist, in which disparate intensive insulin strategies were implemented, and might potential introduce bias and impair the robustness of the conclusions. Fourth, practical difficulties should be noted. When each arm of trials failed to attain the glucose target, it might introduce bias. Finally, as insufficient data of secondary endpoints was provided by enrolled trials, we were unable to assess other sources of bias for several secondary outcomes. Thus, more advanced glucose monitoring devices such as artificial pancreas should urgently be equipped for better outcomes 28.

## Conclusions

The current systematic review and meta-analysis demonstrated that an intensive glucose control strategy among critically ill patients was potentially associated with a reduced risk of all-cause death in comparison to a regular glucose control strategy. Moreover, our results revealed that ICU patients who underwent intensive glucose control did not increase the risk of developing infection and sepsis during hospitalization and that their ICU length of stay was relatively shorter than that of those who received usual care. Consistent with previous studies, we found that the risk of severe hypoglycemia was significantly elevated among patients with intensive glucose control. Our results might indicate the beneficial role of an intensive glucose control strategy in many dimensions, which challenged the current recommendation of glucose control strategy in critically ill patients. We believe that further clinical trials are required to test our findings.

## Supplementary Material

Supplementary figures and tables.

## Figures and Tables

**Table 1:** Characteristics of all eligible randomized control trials comparing intensive glucose control to usual care among critically ill patients.

Author	Year	No. of sites	No. of patients	ICU Setting	Diabetic (%)	Age (years)	Intensive glucose control			Usual care		Follow- up
							Target glucose (mg/dL)	Glucose achieved (mg/dL)		Target glucose (mg/dL)	Glucose achieved (mg/dL)	
Annane 42	2010	8	509	Mixed ICU	N/A	64	80-110	124		180-200	150	6 months
Arabi 43	2008	1	523	Mixed ICU	40%	52.4	80-110	115		180-200	171	Hospital stay
Arabi 44	2011	1	240	Mixed ICU	40%	51.1	80-110	112		180-200	155	6 months
Bilotta 47	2007	1	78	Surgical ICU	10%	52.5	80-120	93		-220	147	6 months
Bilotta 45	2008	1	97	Surgical ICU	12%	52.5	80-120	92		-220	147	6 months
Bilotta 46	2009	1	483	Surgical ICU	10%	57.1	80-110	92		-215	143	6 months
Bland 48	2005	1	10	Medical ICU	40%	56.7	80-110	105		180-200	177	28 days
Brunkhorst 11	2008	18	537	Mixed ICU	30%	64.6	80-110	112		180-200	151	90 days
Bruno 49	2008	5	46	Medical ICU	91%	59.1	90-130	133		-200	190	90 days
Cao 50	2011	1	179	Surgical ICU	100%	58.8	80-110	99		180-198	178	28 days
Cappi 51	2012	1	63	Mixed ICU	24%	53	80-110	99		140-180	155	Hospital stay
Chan 52	2009	1	109	Surgical ICU	29%	57.5	80-130	127		160-200	168	30 days
Coester 53	2010	1	88	Surgical ICU	1%	38.5	80-110	123		-180	145	6 months
Davies 54	1991	4	69	Medical ICU	100%	62	72-144	185		-180	193	Hospital stay
De Azevedo 55	2010	2	337	Mixed ICU	31%	56.2	80-120	134		-180	144	ICU stay
De La Rosa 56	2008	1	504	Mixed ICU	12%	46.6	80-110	117		180-200	149	Hospital stay
Desai 57	2012	1	189	Surgical ICU	43%	62.7	90-120	N/A		121-180	N/A	30 days
Dong 58	2009	1	27	Medical ICU	0%	44	74-110	108		112-150	148	Hospital stay
Farah 59	2007	1	89	Mixed ICU	60%	73.1	110-140	142		140-200	174	28 days
Fernandez^#^ 81	2005	1	20	Medical ICU	85%	71.9	80-110	120		180-200	205	Hospital stay
Finfer 12	2009	42	6104	Mixed ICU	20%	60.2	81-108	118		-180	145	90 days
Gandhi 60	2007	1	371	Surgical ICU	20%	63	80-100	113		-200	157	Hospital stay
Giakoumidakis 61	2013	1	212	Surgical ICU	29%	65.9	120-160	154		161-200	174	Hospital stay
Gray 62	2007	21	933	Surgical ICU	16%	75.2	72-126	113		-306	122	20 days
Green 63	2010	1	81	Mixed ICU	N/A	51	80-110	112		-150	143	90 days
Grey 64	2004	1	61	Surgical ICU	12%	55.6	80-120	125		180-220	179	Hospital stay
He 65	2007	1	188	Mixed ICU	18%	65.5	80-110	108		180-200	180	Hospital stay
Henderson 66	2009	1	67	Mixed ICU	9%	56.5	90-126	113		162-198	151	28 days
Jin^#^ 69	2009	14	356	Medical ICU	N/A	65.7	80-110	99		180-200	189	28 days
Johnston 70	2009	2	74	Medical ICU	60%	68.3	70-110	112		-200	151	90 days
Kalfon 13	2014	34	2684	Mixed ICU	20%	61.5	80-110	115		-180	126	90 days
Kia^#^ 73	2005	1	265	Surgical ICU	26%	68.2	75-115	109		180-200	144	90 days
Kreisel 71	2009	1	40	Medical ICU	33%	71.6	80-110	117		-200	144	120 days
Iapichino 67	2008	3	72	Mixed ICU	17%	62.3	80-110	110		180-200	163	90 days
Lazar 72	2011	1	82	Surgical ICU	100%	64	90-120	103		120-180	135	30 days
Mackenzie^#^ 68	2005	2	240	Mixed ICU	83%	64.5	72-108	126		180-198	151	Hospital stay
Mahmoodpoor 74	2011	1	60	Surgical ICU	15%	52.8	80-120	109		-200	141	ICU stay
McMullin 75	2007	1	20	Mixed ICU	55%	68.8	90-126	128		144-180	169	Hospital stay
Meng 89	2009	1	240	Surgical ICU	10%	46	80-110	N/A		180-200	N/A	6 months
Miranda 76	2013	1	27	Medical ICU	N/A	53.8	80-110	N/A		180-220	N/A	72 hours
Mitchell 77	2006	1	70	Mixed ICU	14%	65.8	80-110	97		180-200	142	Hospital stay
Okabayashi 78	2014	1	502	Surgical ICU	24%	66.5	80-110	106		140-180	155	Hospital stay
Oksanen 79	2007	2	90	Medical ICU	79%	64	72-108	90		108-144	115	30 days
Preiser 80	2009	21	1078	Mixed ICU	19%	64.6	80-110	117		140-180	144	Hospital stay
Rosso 82	2012	1	180	Medical ICU	13%	73.3	-99	103		-144	117	90 days
Savioli 83	2009	3	90	Mixed ICU	13%	61	80-110	112		180-200	159	28 days
Staszewski 84	2011	1	50	Medical ICU	0%	77.1	81-126	108		180-	122	30 days
Stecher^#^ 41	2006	1	117	Surgical ICU	13%	52.6	80-110	N/A		140-180	N/A	N/A
Taslimi 85	2009	1	129	Mixed ICU	53%	55.5	80-110	N/A		125-225	N/A	ICU stay
Van den Berghe 9	2001	1	1548	Surgical ICU	13%	62.8	80-110	103		180-200	153	Hospital stay
Van den Berghe 14	2006	1	1200	Medical ICU	17%	63.5	80-110	111		180-200	153	90 days
Walters 86	2006	1	25	Medical ICU	52%	74.9	90-144	122		-270	135	30 days
Wang 87	2006	1	116	Mixed ICU	11%	65.1	80-110	99		180-200	185	Hospital stay
Wang 88	2017	1	88	Medical ICU	19%	46.7	80-110	N/A		180-200	N/A	6 months
Yang 90	2009	1	110	Medical ICU	N/A	59.7	80-150	160		Treated withtwice daily insulin	229	N/A
Yu 91	2005	1	55	Mixed ICU	N/A	46	80-110	103		180-200	198	Hospital stay
Zhang 92	2008	1	338	Surgical ICU	28%	60.9	80-110	110		130-150	139	Hospital stay

Abbreviations: ICU, intensive care unit; N/A, not available from manuscript or authors. #: Abstract only.

**Table 2:** Subgroup analyses on all-cause mortality

Subgroup		No. of studies	No. of patients	OR (95% CI)	I^2^	*P* value
**Admission category**						
	Medical ICU	16	2295	0.75 (0.59-0.95)	6%	0.02
	Surgical ICU	18	5605	0.83 (0.70-0.99)	1%	0.04
	Mixed ICU	23	13940	0.97 (0.84-1.11)	42%	0.65
**Sepsis and septic shock**						
	Septic patients	13	3107	0.96 (0.73-1.25)	40%	0.74
	Non-septic patients	47	18753	0.89 (0.79-1.01)	33%	0.07
**Neurocritical illness**						
	Neurocritical care patients	19	2800	0.91 (0.75-1.10)	0%	0.32
	Non-neurocritical care patients	42	19050	0.88 (0.77-1.01)	45%	0.08
**Diabetes mellitus**						
	Diabetic patients	8	2217	1.12 (0.91-1.37)	0%	0.28
	Non-diabetic patients	54	19621	0.89 (0.79-1.00)	32%	0.05

Abbreviations: ICU, intensive care unit; OR, odds ratio; CI, confidence interval.

**Table 3:** Summary of primary and secondary outcomes

Outcome		No. of studies	OR (95%CI)	MD (95%CI)	I^2^	*P* value	Quality of evidence^a^
**Primary endpoint**							
	All-cause mortality	57	0.89 (0.80-1.00)		32%	0.04	Low (Inconsistency, Publication bias)
**Secondary endpoints**							
	90-day mortality	11	1.04 (0.95-1.13)		3%	0.39	Moderate (Inconsistency)
	180-day mortality	9	0.99 (0.81-1.20)		0%	0.89	Moderate (Inconsistency)
	ICU mortality	16	0.95 (0.85-1.06)		19%	0.36	Moderate (Inconsistency)
	Severe hypoglycemia	32	5.63 (4.02-7.87)		67%	<0.0001	Moderate (Risk of bias)
	Need for RRT	16	1.07 (0.95-1.20)		0%	0.29	Low (Indirectness, Imprecision)
	Infection	19	0.65 (0.51-0.82)		47%	0.0002	Moderate (Imprecision)
	Sepsis	12	0.80 (0.65-0.99)		0%	0.04	Moderate (Imprecision)
	ICU length of stay	12		-0.70 (-1.21--0.19)	70%	0.007	Low (Inconsistency, Risk of bias)
	Hospital length of stay	8		-1.29 (-2.56--0.01)	61%	0.05	Low (Inconsistency, Risk of bias)

Abbreviations: ICU, intensive care unit; OR, odds ratio; MD, mean difference; CI, confidence interval. ^a^Quality of evidence of each outcome was assessed by using GRADE method.

## Abbreviations

ICU: intensive care unit
RCT: randomized controlled trial
NICE-SUGAR: Normoglycemia in Intensive Care Evaluation and Survival Using Glucose Algorithm Regulation
ADA: American Diabetes Association
GRADE: Grading of Recommendations Assessment, Development and Evaluation
MeSH: Medical Subject Heading
HIRU: Health Information Research Unit
GKI: glucose-insulin-potassium infusions
RRT: renal replacement therapy
TBI: traumatic brain injury
SAH: aneurysmal subarachnoid hemorrhage
ICH: spontaneous intracerebral hemorrhage
ORs: odds ratios
CIs: confidence intervals

## References

[1] Capes SE, Hunt D, Malmberg K, Pathak P, Gerstein HC (2001). Stress hyperglycemia and prognosis of stroke in nondiabetic and diabetic patients: a systematic overview. Stroke.

[2] Gale SC, Sicoutris C, Reilly PM, Schwab CW, Gracias VH (2007). Poor glycemic control is associated with increased mortality in critically ill trauma patients. The American surgeon.

[3] Krinsley JS (2003). Association between hyperglycemia and increased hospital mortality in a heterogeneous population of critically ill patients. Mayo Clinic proceedings.

[4] Sung J, Bochicchio GV, Joshi M, Bochicchio K, Tracy K, Scalea TM (2005). Admission hyperglycemia is predictive of outcome in critically ill trauma patients. The Journal of trauma.

[5] Thompson BT (2008). Glucose control in sepsis. Clinics in chest medicine.

[6] Whitcomb BW, Pradhan EK, Pittas AG, Roghmann MC, Perencevich EN (2005). Impact of admission hyperglycemia on hospital mortality in various intensive care unit populations. Critical care medicine.

[7] Dungan KM, Braithwaite SS, Preiser JC (2009). Stress hyperglycaemia. Lancet.

[8] Egi M, Finfer S, Bellomo R (2011). Glycemic control in the ICU. Chest.

[9] van den Berghe G, Wouters P, Weekers F, Verwaest C, Bruyninckx F, Schetz M (2001). Intensive insulin therapy in critically ill patients. N Engl J Med.

[10] van Hooijdonk RT, Mesotten D, Krinsley JS, Schultz MJ (2016). Sweet Spot: Glucose Control in the Intensive Care Unit. Seminars in respiratory and critical care medicine.

[11] Brunkhorst FM, Engel C, Bloos F, Meier-Hellmann A, Ragaller M, Weiler N (2008). Intensive insulin therapy and pentastarch resuscitation in severe sepsis. N Engl J Med.

[12] Finfer S, Chittock DR, Su SY, Blair D, Foster D, Dhingra V (2009). Intensive versus conventional glucose control in critically ill patients. N Engl J Med.

[13] Kalfon P, Giraudeau B, Ichai C, Guerrini A, Brechot N, Cinotti R (2014). Tight computerized versus conventional glucose control in the ICU: a randomized controlled trial. Intensive care medicine.

[14] Van den Berghe G, Wilmer A, Hermans G, Meersseman W, Wouters PJ, Milants I (2006). Intensive insulin therapy in the medical ICU. N Engl J Med.

[15] Krinsley J, Schultz MJ, Spronk PE, van Braam Houckgeest F, van der Sluijs JP, Melot C (2011). Mild hypoglycemia is strongly associated with increased intensive care unit length of stay. Annals of intensive care.

[16] Egi M, Bellomo R, Stachowski E, French CJ, Hart GK, Taori G (2010). Hypoglycemia and outcome in critically ill patients. Mayo Clinic proceedings.

[17] Finfer S, Liu B, Chittock DR, Norton R, Myburgh JA, McArthur C (2012). Hypoglycemia and risk of death in critically ill patients. N Engl J Med.

[18] Krinsley JS, Egi M, Kiss A, Devendra AN, Schuetz P, Maurer PM (2013). Diabetic status and the relation of the three domains of glycemic control to mortality in critically ill patients: an international multicenter cohort study. Critical care.

[19] Duning T, van den Heuvel I, Dickmann A, Volkert T, Wempe C, Reinholz J (2010). Hypoglycemia aggravates critical illness-induced neurocognitive dysfunction. Diabetes care.

[20] Mesotten D, Gielen M, Sterken C, Claessens K, Hermans G, Vlasselaers D (2012). Neurocognitive development of children 4 years after critical illness and treatment with tight glucose control: a randomized controlled trial. JAMA.

[21] Plummer MP, Deane AM (2016). Dysglycemia and Glucose Control During Sepsis. Clinics in chest medicine.

[22] Vriesendorp TM, DeVries JH, van Santen S, Moeniralam HS, de Jonge E, Roos YB (2006). Evaluation of short-term consequences of hypoglycemia in an intensive care unit. Critical care medicine.

[23] Diabetes Care in the Hospital (2018). Standards of Medical Care in Diabetes-2018. Diabetes care.

[24] Rhodes A, Evans LE, Alhazzani W, Levy MM, Antonelli M, Ferrer R (2017). Surviving Sepsis Campaign: International Guidelines for Management of Sepsis and Septic Shock: 2016. Intensive Care Med.

[25] Griesdale DE, de Souza RJ, van Dam RM, Heyland DK, Cook DJ, Malhotra A (2009). Intensive insulin therapy and mortality among critically ill patients: a meta-analysis including NICE-SUGAR study data. CMAJ.

[26] Ling Y, Li X, Gao X (2012). Intensive versus conventional glucose control in critically ill patients: a meta-analysis of randomized controlled trials. European journal of internal medicine.

[27] Wiener RS, Wiener DC, Larson RJ (2008). Benefits and risks of tight glucose control in critically ill adults: a meta-analysis. JAMA.

[28] Yamada T, Shojima N, Noma H, Yamauchi T, Kadowaki T (2017). Glycemic control, mortality, and hypoglycemia in critically ill patients: a systematic review and network meta-analysis of randomized controlled trials. Intensive care medicine.

[29] Yatabe T, Inoue S, Sakaguchi M, Egi M (2017). The optimal target for acute glycemic control in critically ill patients: a network meta-analysis. Intensive care medicine.

[30] Song F, Zhong LJ, Han L, Xie GH, Xiao C, Zhao B (2014). Intensive insulin therapy for septic patients: a meta-analysis of randomized controlled trials. BioMed research international.

[31] Angus DC, Linde-Zwirble WT, Lidicker J, Clermont G, Carcillo J, Pinsky MR (2001). Epidemiology of severe sepsis in the United States: analysis of incidence, outcome, and associated costs of care. Critical care medicine.

[32] Taylor JH, Beilman GJ (2005). Hyperglycemia in the intensive care unit: no longer just a marker of illness severity. Surgical infections.

[33] Leonidou L, Michalaki M, Leonardou A, Polyzogopoulou E, Fouka K, Gerolymos M (2008). Stress-induced hyperglycemia in patients with severe sepsis: a compromising factor for survival. The American journal of the medical sciences.

[34] Godoy DA, Di Napoli M, Rabinstein AA (2010). Treating hyperglycemia in neurocritical patients: benefits and perils. Neurocrit Care.

[35] Kramer AH, Roberts DJ, Zygun DA (2012). Optimal glycemic control in neurocritical care patients: a systematic review and meta-analysis. Critical care.

[36] Rady MY, Johnson DJ, Patel BM, Larson JS, Helmers RA (2005). Influence of individual characteristics on outcome of glycemic control in intensive care unit patients with or without diabetes mellitus. Mayo Clinic proceedings.

[37] Egi M, Bellomo R, Stachowski E, French CJ, Hart GK, Hegarty C (2008). Blood glucose concentration and outcome of critical illness: the impact of diabetes. Critical care medicine.

[38] Vincent JL, Preiser JC, Sprung CL, Moreno R, Sakr Y (2010). Insulin-treated diabetes is not associated with increased mortality in critically ill patients. Critical care.

[39] Falciglia M, Freyberg RW, Almenoff PL, D'Alessio DA, Render ML (2009). Hyperglycemia-related mortality in critically ill patients varies with admission diagnosis. Critical care medicine.

[40] Van den Berghe G, Wilmer A, Milants I, Wouters PJ, Bouckaert B, Bruyninckx F (2006). Intensive insulin therapy in mixed medical/surgical intensive care units: benefit versus harm. Diabetes.

[41] Stecher A, Steblaj S, Kremzar B, Ivanova E (2006). The influence of normoglycemia on ventilator-associated pneumonia in trauma patients. Paper presented at: European Trauma Congress; May 24-26.

[42] Annane D, Cariou A, Maxime V, Azoulay E, D'Honneur G, Timsit JF (2010). Corticosteroid treatment and intensive insulin therapy for septic shock in adults: a randomized controlled trial. JAMA.

[43] Arabi YM, Dabbagh OC, Tamim HM, Al-Shimemeri AA, Memish ZA, Haddad SH (2008). Intensive versus conventional insulin therapy: a randomized controlled trial in medical and surgical critically ill patients. Critical care medicine.

[44] Arabi YM, Tamim HM, Dhar GS, Al-Dawood A, Al-Sultan M, Sakkijha MH (2011). Permissive underfeeding and intensive insulin therapy in critically ill patients: a randomized controlled trial. The American journal of clinical nutrition.

[45] Bilotta F, Caramia R, Cernak I, Paoloni FP, Doronzio A, Cuzzone V (2008). Intensive insulin therapy after severe traumatic brain injury: a randomized clinical trial. Neurocritical care.

[46] Bilotta F, Caramia R, Paoloni FP, Delfini R, Rosa G (2009). Safety and efficacy of intensive insulin therapy in critical neurosurgical patients. Anesthesiology.

[47] Bilotta F, Spinelli A, Giovannini F, Doronzio A, Delfini R, Rosa G (2007). The effect of intensive insulin therapy on infection rate, vasospasm, neurologic outcome, and mortality in neurointensive care unit after intracranial aneurysm clipping in patients with acute subarachnoid hemorrhage: a randomized prospective pilot trial. Journal of neurosurgical anesthesiology.

[48] Bland DK, Fankhanel Y, Langford E, Lee M, Lee SW, Maloney C (2005). Intensive versus modified conventional control of blood glucose level in medical intensive care patients: a pilot study. American journal of critical care.

[49] Bruno A, Kent TA, Coull BM, Shankar RR, Saha C, Becker KJ (2008). Treatment of hyperglycemia in ischemic stroke (THIS): a randomized pilot trial. Stroke.

[50] Cao SG, Ren JA, Shen B, Chen D, Zhou YB, Li JS (2011). Intensive versus conventional insulin therapy in type 2 diabetes patients undergoing D2 gastrectomy for gastric cancer: a randomized controlled trial. World journal of surgery.

[51] Cappi SB, Noritomi DT, Velasco IT, Curi R, Loureiro TC, Soriano FG (2012). Dyslipidemia: a prospective controlled randomized trial of intensive glycemic control in sepsis. Intensive care medicine.

[52] Chan RP, Galas FR, Hajjar LA, Bello CN, Piccioni MA, Auler JO (2009). Intensive perioperative glucose control does not improve outcomes of patients submitted to open-heart surgery: a randomized controlled trial. Clinics.

[53] Coester A, Neumann CR, Schmidt MI (2010). Intensive insulin therapy in severe traumatic brain injury: a randomized trial. J Trauma.

[54] Davies RR, Newton RW, McNeill GP, Fisher BM, Kesson CM, Pearson D (1991). Metabolic control in diabetic subjects following myocardial infarction: difficulties in improving blood glucose levels by intravenous insulin infusion. Scott Med J.

[55] de Azevedo JR, de Araujo LO, da Silva WS, de Azevedo RP (2010). A carbohydrate-restrictive strategy is safer and as efficient as intensive insulin therapy in critically ill patients. Journal of critical care.

[56] De La Rosa Gdel C, Donado JH, Restrepo AH, Quintero AM, Gonzalez LG, Saldarriaga NE (2008). Strict glycaemic control in patients hospitalised in a mixed medical and surgical intensive care unit: a randomised clinical trial. Critical care.

[57] Desai SP, Henry LL, Holmes SD, Hunt SL, Martin CT, Hebsur S (2012). Strict versus liberal target range for perioperative glucose in patients undergoing coronary artery bypass grafting: a prospective randomized controlled trial. The Journal of thoracic and cardiovascular surgery.

[58] Dong SM, Qin YJ, Gao YF (2009). The influence of intensive insulin therapy on hemodynamics in patients with septic shock. Zhongguo wei zhong bing ji jiu yi xue.

[59] Farah R, Samokhvalov A, Zviebel F, Makhoul N (2007). Insulin therapy of hyperglycemia in intensive care. Isr Med Assoc J.

[60] Gandhi GY, Nuttall GA, Abel MD, Mullany CJ, Schaff HV, O'Brien PC (2007). Intensive intraoperative insulin therapy versus conventional glucose management during cardiac surgery: a randomized trial. Ann Intern Med.

[61] Giakoumidakis K, Eltheni R, Patelarou E, Theologou S, Patris V, Michopanou N (2013). Effects of intensive glycemic control on outcomes of cardiac surgery. Heart lung.

[62] Gray CS, Hildreth AJ, Sandercock PA, O'Connell JE, Johnston DE, Cartlidge NE (2007). Glucose-potassium-insulin infusions in the management of post-stroke hyperglycaemia: the UK Glucose Insulin in Stroke Trial (GIST-UK). Lancet Neurol.

[63] Green DM, O'Phelan KH, Bassin SL, Chang CW, Stern TS, Asai SM (2010). Intensive versus conventional insulin therapy in critically ill neurologic patients. Neurocritical care.

[64] Grey NJ, Perdrizet GA (2004). Reduction of nosocomial infections in the surgical intensive-care unit by strict glycemic control. Endocrine practice.

[65] He W, Zhang TY, Zhou H, Li T, Zhao JY, Zhao D (2007). Impact of intensive insulin therapy on surgical critically ill patients. Zhonghua wai ke za zhi.

[66] Henderson WR, Dhingra V, Chittock D, Foster D, Hebert P, Cook D (2009). The efficacy and safety of glucose control algorithms in intensive care: a pilot study of the Survival Using Glucose Algorithm Regulation (SUGAR) trial. Polskie Archiwum Medycyny Wewnetrznej.

[67] Iapichino G, Albicini M, Umbrello M, Sacconi F, Fermo I, Pavlovich R (2008). Tight glycemic control does not affect asymmetric-dimethylarginine in septic patients. Intensive care medicine.

[68] Mackenzie IM, Ingle S, Underwood C, Blunt M (2005). Glycaemic control and outcome in general intensive care. Proc Am Thorac Soc.

[69] Jin (2009). Y, Guolong. C. A multicentre study on intensive insulin therapy of severe sepsis and septic shock patients in ICU collaborative study group on IIT in Zhejiang province, china. Intensive Care Med.

[70] Johnston KC, Hall CE, Kissela BM, Bleck TP, Conaway MR (2009). Glucose Regulation in Acute Stroke Patients (GRASP) trial: a randomized pilot trial. Stroke.

[71] Kreisel SH, Berschin UM, Hammes HP, Leweling H, Bertsch T, Hennerici MG (2009). Pragmatic management of hyperglycaemia in acute ischaemic stroke: safety and feasibility of intensive intravenous insulin treatment. Cerebrovascular diseases.

[72] Lazar HL, McDonnell MM, Chipkin S, Fitzgerald C, Bliss C, Cabral H (2011). Effects of aggressive versus moderate glycemic control on clinical outcomes in diabetic coronary artery bypass graft patients. Annals of surgery.

[73] M K, J. B, KR. B. The effects of strict glycemic control in the critically ill general and vascular surgical patient. 91st Annual Clinical Congress of the American College of Surgeons; October 16-20; San Francisco, California2005.

[74] Mahmoodpoor A, Ali-Asgharzadeh A, Parish M, Amir-Aslanzadeh Z, Abedini N (2011). A comparative study of efficacy of intensive insulin therapy versus conventional method on mortality and morbidity of critically ill patients. Pakistan journal of medical sciences.

[75] McMullin J, Brozek J, McDonald E, Clarke F, Jaeschke R, Heels-Ansdell D (2007). Lowering of glucose in critical care: a randomized pilot trial. Journal of critical care.

[76] Miranda MP, Crespo JC, Secoli SR (2013). Insulin infusion in intensive care: randomized controlled trial. Rev Esc Enferm USP.

[77] Mitchell I, Knight E, Gissane J, Tamhane R, Kolli R, Leditschke IA (2006). A phase II randomised controlled trial of intensive insulin therapy in general intensive care patients. Critical care and resuscitation.

[78] Okabayashi T, Shima Y, Sumiyoshi T, Kozuki A, Tokumaru T, Iiyama T (2014). Intensive versus intermediate glucose control in surgical intensive care unit patients. Diabetes care.

[79] Oksanen T, Skrifvars MB, Varpula T, Kuitunen A, Pettilä V, Nurmi J (2007). Strict versus moderate glucose control after resuscitation from ventricular fibrillation. Intensive care medicine.

[80] Preiser JC, Devos P, Ruiz-Santana S, Melot C, Annane D, Groeneveld J (2009). A prospective randomised multi-centre controlled trial on tight glucose control by intensive insulin therapy in adult intensive care units: the Glucontrol study. Intensive care medicine.

[81] Fernandez R, Boque M, Galera A, Rodriguez-Cintron W (2005). Insulin: effect on mortality and renal failure in medical intensive care unit patients. Proc Am Thorac Soc.

[82] Rosso C, Corvol JC, Pires C, Crozier S, Attal Y, Jacqueminet S (2012). Intensive versus subcutaneous insulin in patients with hyperacute stroke: results from the randomized INSULINFARCT trial. Stroke.

[83] Savioli M, Cugno M, Polli F, Taccone P, Bellani G, Spanu P (2009). Tight glycemic control may favor fibrinolysis in patients with sepsis. Critical care medicine.

[84] Staszewski J, Brodacki B, Kotowicz J, Stepien A (2011). Intravenous insulin therapy in the maintenance of strict glycemic control in nondiabetic acute stroke patients with mild hyperglycemia. Journal of stroke and cerebrovascular diseases.

[85] Taslimi R, Azizkhani R, Talebian MH, Abtahi HR, Jalili M, Nejati A (2009). The efficacy of intensive glucose management on hospitalized critically ill patients associated mortality rate in intensive care unit. Daru.

[86] Walters MR, Weir CJ, Lees KR (2006). A randomised, controlled pilot study to investigate the potential benefit of intervention with insulin in hyperglycaemic acute ischaemic stroke patients. Cerebrovascular diseases.

[87] Wang LC, Lei S, Wu YC, Wu JN, Wang LF, Guan TR (2006). Intensive insulin therapy in critically ill patients. Zhongguo wei zhong bing ji jiu yi xue.

[88] Wang Y, Li JP, Song YL, Zhao QH (2017). Intensive insulin therapy for preventing postoperative infection in patients with traumatic brain injury: a randomized controlled trial. Medicine.

[89] Yang M, Guo Q, Zhang X, Sun S, Wang Y, Zhao L (2009). Intensive insulin therapy on infection rate, days in NICU, in-hospital mortality and neurological outcome in severe traumatic brain injury patients: a randomized controlled trial. International journal of nursing studies.

[90] Yang ZL, Liu WD, Ding Y, Liu XY, Zhang YY, Niu HY (2009). Comatose stroke patients complicated with hyperglycemia: A study of realtime insulin titration model. Chinese Journal of Cerebrovascular Diseases.

[91] Yu WK, Li WQ, Wang XD, Yan XW, Qi XP, Li N (2005). Influence and mechanism of a tight control of blood glucose by intensive insulin therapy on human sepsis. Zhonghua wai ke za zhi.

[92] Zhang RL, He W, Li T, Zhou H, Wang C, Gao S (2008). Evaluation of optimal goal of glucose control in critically ill patients. Chinese journal of clinical nutrition.

[93] Waeschle RM, Moerer O, Hilgers R, Herrmann P, Neumann P, Quintel M (2008). The impact of the severity of sepsis on the risk of hypoglycaemia and glycaemic variability. Critical care.

[94] Ali NA, O'Brien JM Jr, Dungan K, Phillips G, Marsh CB, Lemeshow S (2008). Glucose variability and mortality in patients with sepsis. Critical care medicine.

[95] Hirasawa H, Oda S, Nakamura M (2009). Blood glucose control in patients with severe sepsis and septic shock. World journal of gastroenterology.

[96] Monnier L, Mas E, Ginet C, Michel F, Villon L, Cristol JP (2006). Activation of oxidative stress by acute glucose fluctuations compared with sustained chronic hyperglycemia in patients with type 2 diabetes. JAMA.

[97] Clement S, Braithwaite SS, Magee MF, Ahmann A, Smith EP, Schafer RG (2004). Management of diabetes and hyperglycemia in hospitals. Diabetes care.

[98] Kwoun MO, Ling PR, Lydon E, Imrich A, Qu Z, Palombo J (1997). Immunologic effects of acute hyperglycemia in nondiabetic rats. JPEN.

[99] Ceriello A, Novials A, Ortega E, La Sala L, Pujadas G, Testa R (2012). Evidence that hyperglycemia after recovery from hypoglycemia worsens endothelial function and increases oxidative stress and inflammation in healthy control subjects and subjects with type 1 diabetes. Diabetes.

[100] Ceriello A, Novials A, Ortega E, Pujadas G, La Sala L, Testa R (2014). Hyperglycemia following recovery from hypoglycemia worsens endothelial damage and thrombosis activation in type 1 diabetes and in healthy controls. Nutrition, metabolism, and cardiovascular diseases: NMCD.

[101] Suh SW, Gum ET, Hamby AM, Chan PH, Swanson RA (2007). Hypoglycemic neuronal death is triggered by glucose reperfusion and activation of neuronal NADPH oxidase. The Journal of clinical investigation.

