# Supplementary material for: Is intensive glucose control bad for critically ill patients? A systematic review and meta-analysis
Source: Int J Biol Sci. 2020 Mar 12;16(9):1658–75. doi: 10.7150/ijbs.43447 (PMC7097913; doi:10.7150/ijbs.43447)
Supplement: Supplementary file 1 — Supplementary figures and tables. [file ijbsv16p1658s1.pdf]

Supplementary Figure S1.

Study or Subgroup	intensive control Events	intensive control Total	usual care Events	usual care Total	Weight	Odds Ratio M-H, Fixed, 95% CI
Annane2010	122	255	114	254	5.5%	1.13 [0.79, 1.60]
Brunkhorst2008	98	247	102	288	5.2%	1.20 [0.84, 1.70]
Bruno2008	2	31	0	15	0.1%	2.63 [0.12, 58.20]
Finfer2009	670	3010	627	3012	44.7%	1.09 [0.96, 1.23]
Green2010	16	45	9	36	0.6%	1.66 [0.63, 4.37]
Johnston2009	3	24	1	24	0.1%	3.29 [0.32, 34.08]
Kalfon2014	431	1336	447	1312	28.0%	0.92 [0.78, 1.08]
Kia2005	19	132	11	133	0.9%	1.86 [0.85, 4.09]
Lapichino2008	13	36	11	36	0.6%	1.28 [0.48, 3.43]
Rosso2012	9	90	14	90	1.2%	0.60 [0.25, 1.47]
Van den Berghe2006	214	595	228	605	13.3%	0.93 [0.73, 1.17]
**Total (95% CI)**		**5801**		**5805**	**100.0%**	**1.04 [0.95, 1.13]**
Total events	1597		1564			

Heterogeneity: Chi² = 10.29, df = 10 (P = 0.42); I² = 3%
Test for overall effect: Z = 0.86 (P = 0.39)

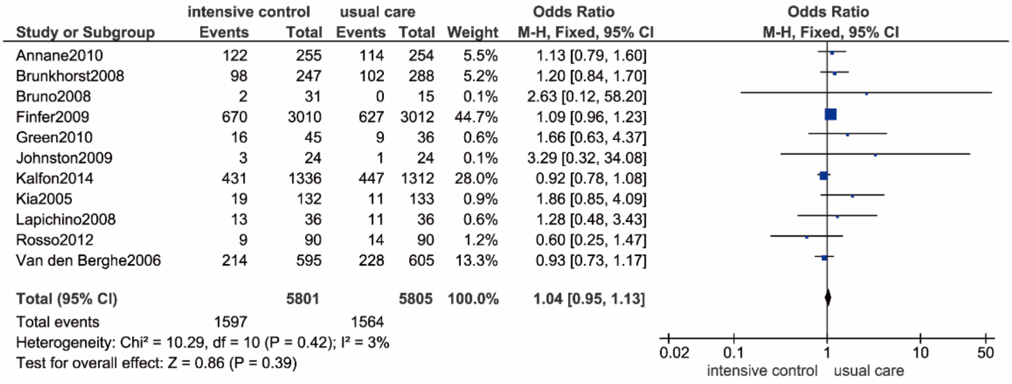

Supplementary Figure S2.

Study or Subgroup	intensive control Events	intensive control Total	usual care Events	usual care Total	Weight	Odds Ratio M-H, Fixed, 95% CI
Annane2010	125	255	121	254	31.3%	1.06 [0.75, 1.50]
Arabi2011	45	118	45	115	14.3%	0.96 [0.57, 1.63]
Bilotta2007	6	40	7	38	3.1%	0.78 [0.24, 2.58]
Bilotta2008	5	48	6	49	2.7%	0.83 [0.24, 2.94]
Bilotta2009	62	241	67	242	25.2%	0.90 [0.60, 1.35]
Coester2010	11	39	11	40	4.0%	1.04 [0.39, 2.77]
Kreisel2009	4	20	2	20	0.8%	2.25 [0.36, 13.97]
Meng2009	61	117	62	116	15.1%	0.95 [0.57, 1.59]
Wang2017	10	44	9	44	3.5%	1.14 [0.41, 3.16]
**Total (95% CI)**		**922**		**918**	**100.0%**	**0.99 [0.81, 1.20]**
Total events	329		330			

Heterogeneity: Chi² = 1.45, df = 8 (P = 0.99); I² = 0%
Test for overall effect: Z = 0.14 (P = 0.89)

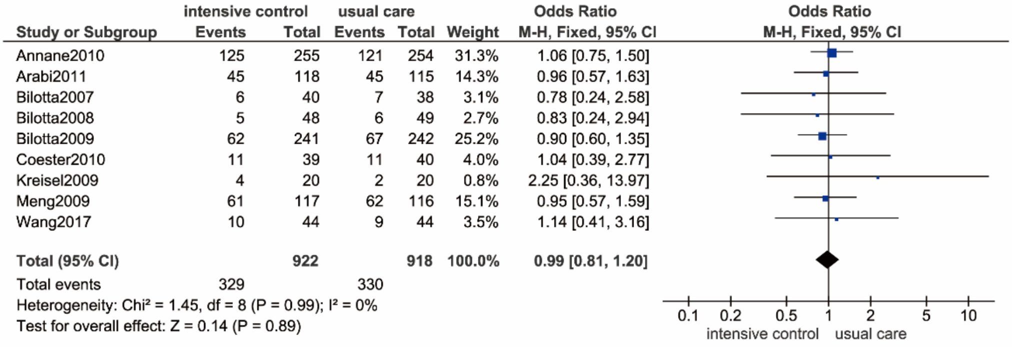

Supplementary Figure S3.

Study or Subgroup	intensive control Events	intensive control Total	usual care Events	usual care Total	Weight	Odds Ratio M-H, Fixed, 95% CI
Arabi2008	18	55	15	67	1.4%	1.69 [0.75, 3.77]
Arabi2011	21	120	26	120	3.3%	0.77 [0.40, 1.46]
Coester2010	9	39	7	40	0.8%	1.41 [0.47, 4.27]
De Azevedo2010	38	168	42	169	5.0%	0.88 [0.53, 1.46]
De La Rosa2008	84	254	78	250	8.1%	1.09 [0.75, 1.58]
Farah2007	16	41	16	48	1.4%	1.28 [0.54, 3.05]
Kalfon2014	302	1336	310	1312	37.0%	0.94 [0.79, 1.13]
Lapichino2008	8	36	6	36	0.7%	1.43 [0.44, 4.64]
Mahmoodpoor2011	6	30	8	30	1.0%	0.69 [0.21, 2.30]
McMullin2007	2	11	4	9	0.6%	0.28 [0.04, 2.09]
Mitchell2006	7	35	2	35	0.2%	4.13 [0.79, 21.48]
Preiser2009	92	536	83	542	10.5%	1.15 [0.83, 1.58]
Taslimi2009	26	67	19	62	1.8%	1.44 [0.69, 2.98]
Van den Berghe2001	35	765	63	783	9.1%	0.55 [0.36, 0.84]
Van den Berghe2006	144	595	162	605	18.6%	0.87 [0.67, 1.13]
Yu2005	3	28	4	27	0.6%	0.69 [0.14, 3.42]
**Total (95% CI)**		**4116**		**4135**	**100.0%**	**0.95 [0.85, 1.06]**
Total events	811		845			

Heterogeneity: Chi² = 18.62, df = 15 (P = 0.23); I² = 19%
Test for overall effect: Z = 0.91 (P = 0.36)

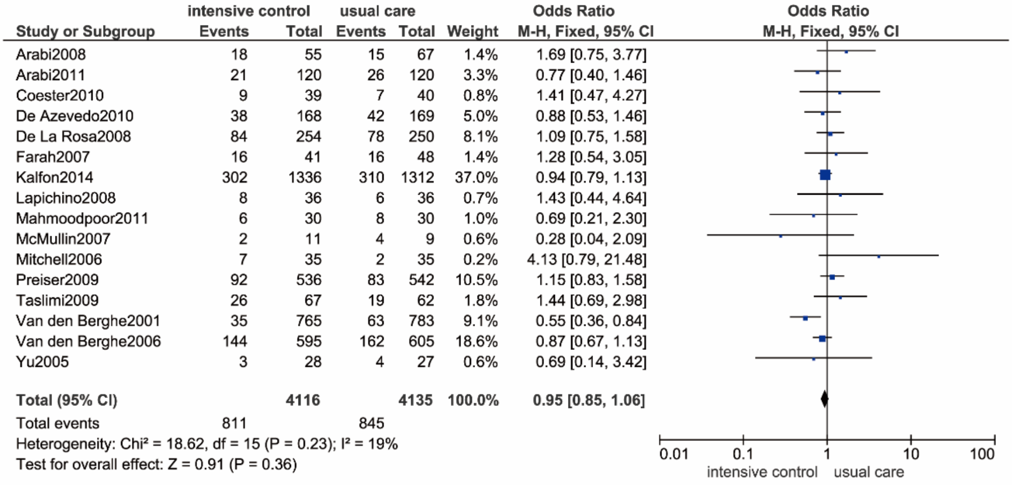

Supplementary Figure S4.

Study or Subgroup	intensive control Events	intensive control Total	usual care Events	usual care Total	Weight	Odds Ratio M-H, Random, 95% CI
**1.9.1 Medical ICU**						
Bland2005	1	5	1	5	2.0%	1.00 [0.05, 22.18]
Bruno2008	11	31	0	15	2.3%	17.39 [0.95, 318.36]
Dong2009	5	13	4	14	7.6%	1.56 [0.31, 7.82]
Kreisel2009	7	20	1	20	4.0%	10.23 [1.12, 93.34]
Oksanen2007	0	39	0	51		Not estimable
Rosso2012	5	90	0	90	2.3%	11.64 [0.63, 213.76]
Staszewski2011	2	26	0	24	2.1%	5.00 [0.23, 109.62]
Van den Berghe2006	111	595	19	605	77.9%	7.07 [4.28, 11.68]
Walters2006	1	13	0	12	1.8%	3.00 [0.11, 80.95]
**Subtotal (95% CI)**		**832**		**836**	**100.0%**	**6.22 [3.99, 9.67]**
Total events	143		25			
Heterogeneity: Tau² = 0.00; Chi² = 5.51, df = 7 (P = 0.60); I² = 0%						
Test for overall effect: Z = 8.09 (P < 0.00001)						
**1.9.2 Surgical ICU**						
Bilotta2009	226	241	152	242	31.7%	8.92 [4.97, 16.00]
Coester2010	6	39	0	40	6.3%	15.72 [0.85, 289.26]
Giakoumidakis2013	0	105	0	107		Not estimable
Lazar2011	30	40	4	42	19.6%	28.50 [8.13, 99.92]
Meng2009	4	121	3	119	16.0%	1.32 [0.29, 6.04]
Van den Berghe2001	39	765	6	783	26.4%	6.96 [2.93, 16.53]
**Subtotal (95% CI)**		**1311**		**1333**	**100.0%**	**8.01 [3.61, 17.79]**
Total events	305		165			
Heterogeneity: Tau² = 0.43; Chi² = 9.74, df = 4 (P = 0.05); I² = 59%						
Test for overall effect: Z = 5.11 (P < 0.00001)						
**1.9.3 Mixed ICU**						
Annane2010	42	255	20	254	8.8%	2.31 [1.31, 4.05]
Arabi2008	76	266	8	257	7.8%	12.45 [5.87, 26.42]
Arabi2011	38	120	8	120	7.5%	6.49 [2.87, 14.64]
Brunkhorst2008	42	247	12	290	8.3%	4.75 [2.44, 9.24]
Cappi2012	2	28	2	35	3.1%	1.27 [0.17, 9.63]
De Azevedo2010	27	168	6	169	7.0%	5.20 [2.09, 12.96]
De La Rosa2008	21	254	2	250	4.7%	11.18 [2.59, 48.19]
Finfer2009	206	3016	15	3014	8.9%	14.66 [8.66, 24.82]
Green2010	2	45	0	36	1.7%	4.20 [0.20, 90.20]
Henderson2009	7	32	1	35	2.9%	9.52 [1.10, 82.38]
Kalfon2014	174	1317	79	1284	9.9%	2.32 [1.76, 3.07]
Lapichino2008	8	36	3	36	4.9%	3.14 [0.76, 12.99]
Mackenzie2005	50	121	9	119	7.8%	8.61 [3.99, 18.59]
McMullin2007	4	11	1	9	2.4%	4.57 [0.41, 51.14]
Mitchell2006	5	35	0	35	1.8%	12.80 [0.68, 241.03]
Preiser2009	44	536	13	542	8.4%	3.64 [1.94, 6.84]
Wang2006	6	58	2	58	4.1%	3.23 [0.62, 16.73]
**Subtotal (95% CI)**		**6545**		**6543**	**100.0%**	**5.21 [3.39, 8.02]**
Total events	754		181			
Heterogeneity: Tau² = 0.47; Chi² = 63.51, df = 16 (P < 0.00001); I² = 75%						
Test for overall effect: Z = 7.51 (P < 0.00001)						

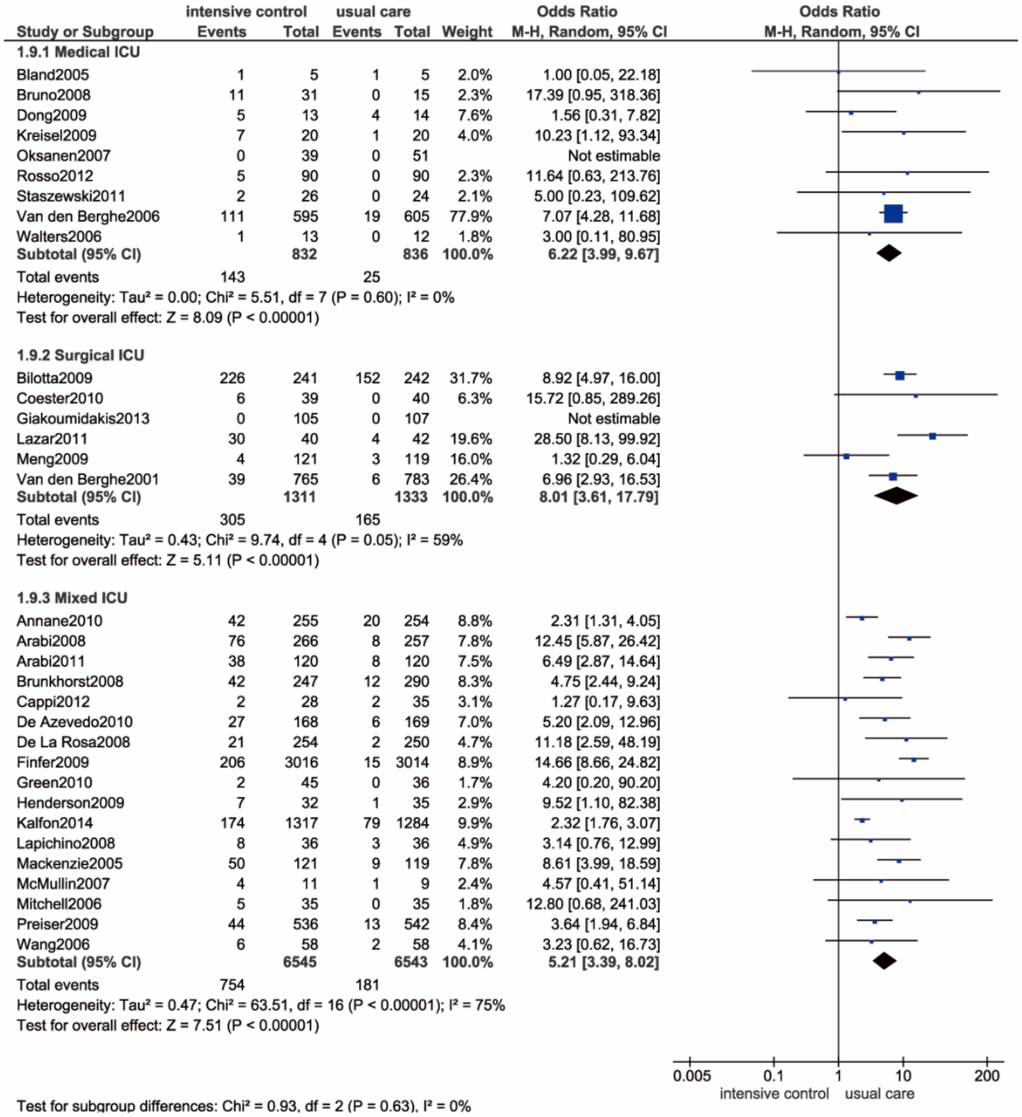

Test for subgroup differences: Chi² = 0.93, df = 2 (P = 0.63), I² = 0%

Supplementary Figure S5.

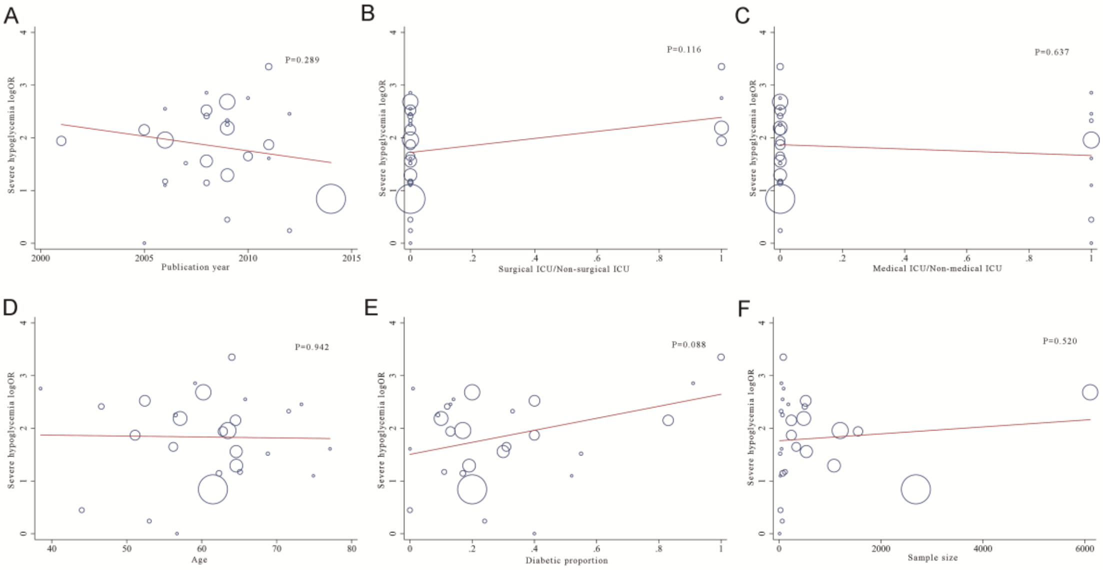

Supplementary Figure S6.

Study or Subgroup	intensive control Events	intensive control Total	usual care Events	usual care Total	Weight	Odds Ratio M-H, Fixed, 95% CI
Arabi2008	31	266	31	257	5.1%	0.96 [0.57, 1.63]
Arabi2011	20	120	18	120	2.8%	1.13 [0.57, 2.27]
Brunkhorst2008	76	244	77	289	8.9%	1.25 [0.86, 1.81]
Cappi2012	7	28	9	35	1.1%	0.96 [0.31, 3.02]
Chan2009	1	54	5	55	0.9%	0.19 [0.02, 1.67]
Coester2010	3	39	5	40	0.8%	0.58 [0.13, 2.63]
De Azevedo2010	16	168	17	169	2.8%	0.94 [0.46, 1.93]
De La Rosa2008	32	254	25	250	4.1%	1.30 [0.74, 2.26]
Desai2012	2	91	0	98	0.1%	5.50 [0.26, 116.17]
Farah2007	22	41	31	48	2.4%	0.63 [0.27, 1.49]
Finfer2009	465	3014	438	3014	68.2%	1.07 [0.93, 1.24]
Gandhi2007	6	185	4	186	0.7%	1.53 [0.42, 5.50]
Henderson2009	2	32	2	35	0.3%	1.10 [0.15, 8.30]
Mahmoodpoor2011	2	30	4	30	0.7%	0.46 [0.08, 2.75]
McMullin2007	0	11	1	9	0.3%	0.25 [0.01, 6.82]
Yu2005	4	28	4	27	0.6%	0.96 [0.21, 4.29]
**Total (95% CI)**		**4605**		**4662**	**100.0%**	**1.07 [0.95, 1.20]**
Total events	689		671			

Heterogeneity: Chi² = 8.94, df = 15 (P = 0.88); I² = 0%

Test for overall effect: Z = 1.06 (P = 0.29)

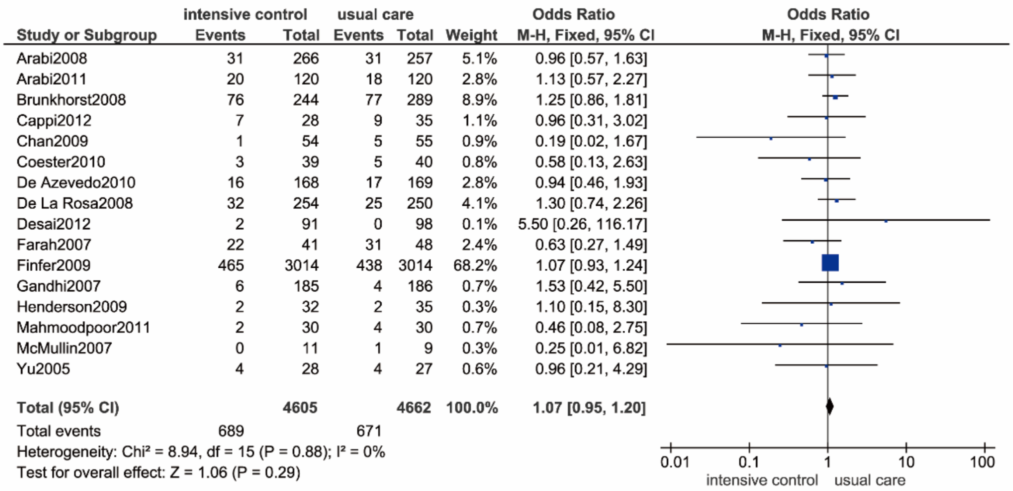

Supplementary Figure S7.

Study or Subgroup	intensive control Events	intensive control Total	usual care Events	usual care Total	Weight	Odds Ratio M-H, Random, 95% CI
Annane2010	47	255	43	254	8.9%	1.11 [0.70, 1.75]
Arabi2008	56	266	59	257	9.4%	0.89 [0.59, 1.35]
Bilotta2007	11	40	16	38	4.2%	0.52 [0.20, 1.34]
Bilotta2008	12	48	19	49	4.7%	0.53 [0.22, 1.26]
Bilotta2009	62	241	95	242	9.8%	0.54 [0.36, 0.79]
Cao2011	7	92	16	87	4.2%	0.37 [0.14, 0.94]
Chan2009	9	54	18	55	4.4%	0.41 [0.17, 1.02]
De Azevedo2010	82	168	84	169	9.3%	0.96 [0.63, 1.48]
De La Rosa2008	69	254	83	250	9.9%	0.75 [0.51, 1.10]
Desai2012	3	91	0	98	0.6%	7.79 [0.40, 152.94]
Farah2007	30	41	38	48	4.0%	0.72 [0.27, 1.91]
Giakoumidakis2013	9	105	12	107	4.4%	0.74 [0.30, 1.84]
Green2010	33	45	24	36	4.1%	1.38 [0.53, 3.58]
He2007	10	75	16	38	4.3%	0.21 [0.08, 0.53]
Henderson2009	4	32	17	35	2.8%	0.15 [0.04, 0.52]
McMullin2007	3	11	2	9	1.2%	1.31 [0.17, 10.26]
Meng2009	38	121	55	119	8.0%	0.53 [0.31, 0.90]
Wang2017	13	44	23	44	4.6%	0.38 [0.16, 0.92]
Zhang2008	7	10	7	12	1.5%	1.67 [0.28, 9.82]
**Total (95% CI)**		**1993**		**1947**	**100.0%**	**0.65 [0.51, 0.82]**
Total events	505		627			

Heterogeneity: Tau² = 0.10; Chi² = 34.13, df = 18 (P = 0.01); I² = 47%

Test for overall effect: Z = 3.69 (P = 0.0002)

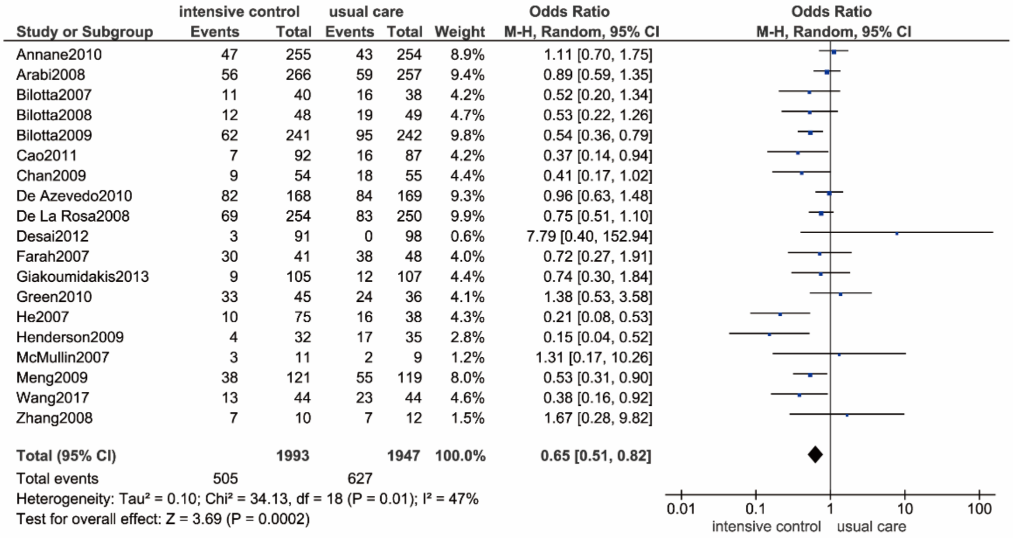

Supplementary Figure S8.

Study or Subgroup	intensive control Events	intensive control Total	usual care Events	usual care Total	Weight	Odds Ratio M-H, Fixed, 95% CI
Arabi2008	98	266	105	257	34.2%	0.84 [0.59, 1.20]
Arabi2011	59	120	50	120	12.9%	1.35 [0.81, 2.25]
Bilotta2008	2	48	3	49	1.4%	0.67 [0.11, 4.18]
Bilotta2009	7	241	8	242	3.9%	0.88 [0.31, 2.45]
Cao2011	2	92	3	87	1.5%	0.62 [0.10, 3.82]
Chan2009	2	54	2	55	1.0%	1.02 [0.14, 7.51]
Coester2010	33	39	32	40	2.5%	1.38 [0.43, 4.41]
Farah2007	11	41	17	48	5.8%	0.67 [0.27, 1.66]
Mahmoodpoor2011	7	30	9	30	3.5%	0.71 [0.22, 2.25]
Meng2009	5	121	7	119	3.4%	0.69 [0.21, 2.24]
Van den Berghe2001	32	765	61	783	29.3%	0.52 [0.33, 0.80]
Wang2017	1	44	1	44	0.5%	1.00 [0.06, 16.51]
**Total (95% CI)**		**1861**		**1874**	**100.0%**	**0.80 [0.65, 0.99]**
Total events	259		298			

Heterogeneity: Chi² = 9.27, df = 11 (P = 0.60); I² = 0%

Test for overall effect: Z = 2.04 (P = 0.04)

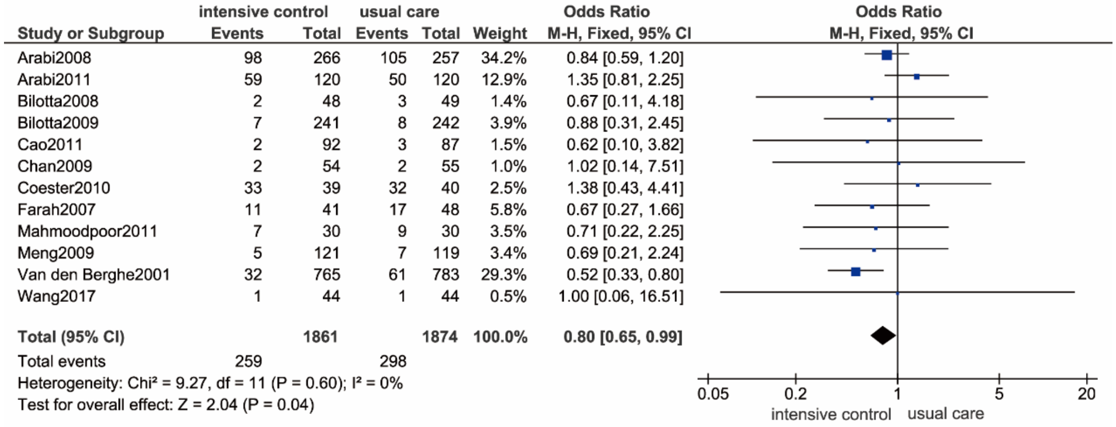

Supplementary Figure S9.

Study or Subgroup	intensive control Mean	intensive control SD	intensive control Total	usual care Mean	usual care SD	usual care Total	Weight	Mean Difference IV, Random, 95% CI
Arabi2008	9.6	8.5	266	10.8	11.3	257	6.0%	-1.20 [-2.92, 0.52]
Arabi2011	13.1	9.8	120	13.1	14.7	120	2.3%	0.00 [-3.16, 3.16]
Chan2009	4.1	6.9	54	5.9	10.2	55	2.2%	-1.80 [-5.06, 1.46]
Coester2010	18.2	27.6	39	12.9	12.7	40	0.3%	5.30 [-4.21, 14.81]
Farah2007	7	4.9	41	8	4.85	48	4.7%	-1.00 [-3.03, 1.03]
Gandhi2007	2	2	185	2	3	186	15.9%	0.00 [-0.52, 0.52]
Giakoumidakis2013	2.7	2.5	105	3.3	4.2	107	11.7%	-0.60 [-1.53, 0.33]
Jin2009	13.9	1.7	115	14.4	1.54	131	16.9%	-0.50 [-0.91, -0.09]
Lazar2011	2.9	0.7	40	2.7	0.5	42	18.1%	0.20 [-0.06, 0.46]
Taslimi2009	7.87	4.01	67	10.19	3.99	62	8.0%	-2.32 [-3.70, -0.94]
Wang2006	9.14	5.45	58	12.88	8.29	58	3.3%	-3.74 [-6.29, -1.19]
Wang2017	4.5	2.1	44	5.7	2.8	44	10.7%	-1.20 [-2.23, -0.17]
**Total (95% CI)**			**1134**			**1150**	**100.0%**	**-0.70 [-1.21, -0.19]**

Heterogeneity: Tau² = 0.36; Chi² = 36.16, df = 11 (P = 0.0002); I² = 70%

Test for overall effect: Z = 2.68 (P = 0.007)

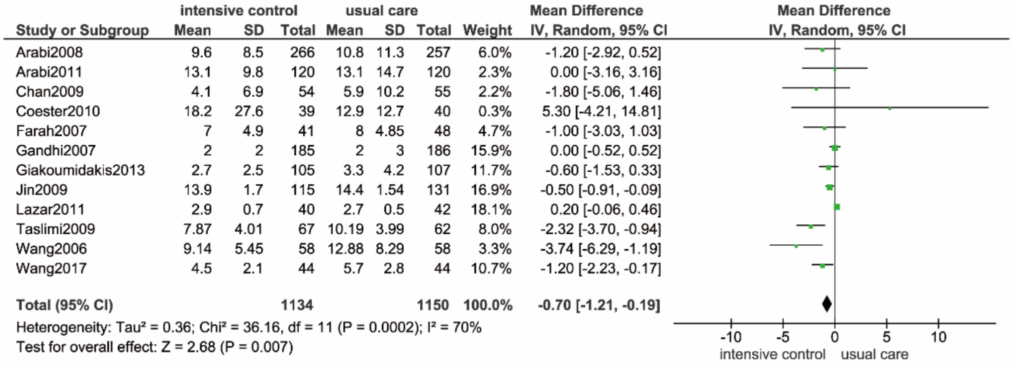

Supplementary Figure S10.

Study or Subgroup	intensive control Mean	SD	Total	usual care Mean	SD	Total	Weight	Mean Difference IV, Random, 95% CI
**1.14.1 Medical ICU**								
Jin2009	13.9	1.7	115	14.4	1.54	131	16.9%	-0.50 [-0.91, -0.09]
Wang2017	4.5	2.1	44	5.7	2.8	44	10.7%	-1.20 [-2.23, -0.17]
**Subtotal (95% CI)**			**159**			**175**	**27.6%**	**-0.68 [-1.28, -0.08]**
Heterogeneity: Tau² = 0.08; Chi² = 1.52, df = 1 (P = 0.22); I² = 34%								
Test for overall effect: Z = 2.22 (P = 0.03)								
**1.14.2 Surgical ICU**								
Chan2009	4.1	6.9	54	5.9	10.2	55	2.2%	-1.80 [-5.06, 1.46]
Coester2010	18.2	27.6	39	12.9	12.7	40	0.3%	5.30 [-4.21, 14.81]
Gandhi2007	2	2	185	2	3	186	15.9%	0.00 [-0.52, 0.52]
Giakoumidakis2013	2.7	2.5	105	3.3	4.2	107	11.7%	-0.60 [-1.53, 0.33]
Lazar2011	2.9	0.7	40	2.7	0.5	42	18.1%	0.20 [-0.06, 0.46]
**Subtotal (95% CI)**			**423**			**430**	**48.1%**	**0.02 [-0.34, 0.38]**
Heterogeneity: Tau² = 0.04; Chi² = 5.32, df = 4 (P = 0.26); I² = 25%								
Test for overall effect: Z = 0.11 (P = 0.91)								
**1.14.3 Mixed ICU**								
Arabi2008	9.6	8.5	266	10.8	11.3	257	6.0%	-1.20 [-2.92, 0.52]
Arabi2011	13.1	9.8	120	13.1	14.7	120	2.3%	0.00 [-3.16, 3.16]
Farah2007	7	4.9	41	8	4.85	48	4.7%	-1.00 [-3.03, 1.03]
Taslimi2009	7.87	4.01	67	10.19	3.99	62	8.0%	-2.32 [-3.70, -0.94]
Wang2006	9.14	5.45	58	12.88	8.29	58	3.3%	-3.74 [-6.29, -1.19]
**Subtotal (95% CI)**			**552**			**545**	**24.3%**	**-1.76 [-2.76, -0.76]**
Heterogeneity: Tau² = 0.28; Chi² = 5.07, df = 4 (P = 0.28); I² = 21%								
Test for overall effect: Z = 3.46 (P = 0.0005)								
**Total (95% CI)**			**1134**			**1150**	**100.0%**	**-0.70 [-1.21, -0.19]**
Heterogeneity: Tau² = 0.36; Chi² = 36.16, df = 11 (P = 0.0002); I² = 70%								
Test for overall effect: Z = 2.68 (P = 0.007)								
Test for subgroup differences: Chi² = 12.76, df = 2 (P = 0.002), I² = 84.3%								

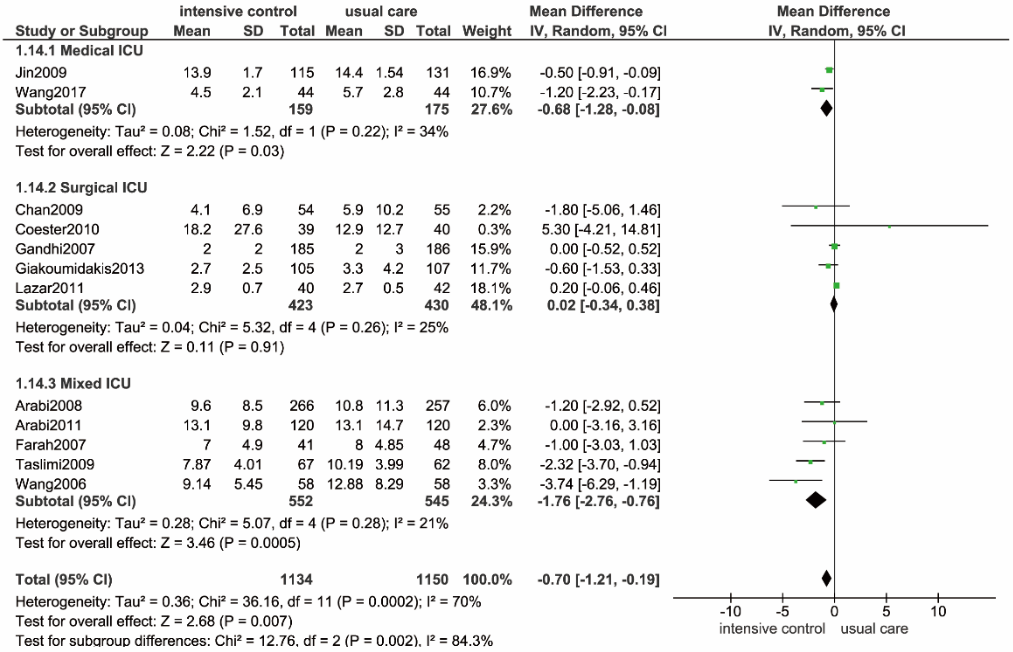

Supplementary Figure S11.

Study or Subgroup	intensive control Mean	SD	Total	usual care Mean	SD	Total	Weight	Mean Difference IV, Random, 95% CI
Arabi2008	54.1	84.1	266	57.5	77.1	257	0.8%	-3.40 [-17.22, 10.42]
Arabi2011	70.7	106.3	120	66.7	94.3	120	0.2%	4.00 [-21.42, 29.42]
Chan2009	12	7	54	17	16	55	5.9%	-5.00 [-9.62, -0.38]
Gandhi2007	8	4	185	8	5	186	23.9%	0.00 [-0.92, 0.92]
Giakoumidakis2013	9.9	7.4	105	9.4	5	107	18.3%	0.50 [-1.20, 2.20]
Lazar2011	10.1	3.5	40	10.8	3.5	42	19.7%	-0.70 [-2.22, 0.82]
Okabayashi2014	18.2	15.8	222	23	16.6	225	10.8%	-4.80 [-7.80, -1.80]
Staszewski2011	14	2	26	16	3	24	20.3%	-2.00 [-3.43, -0.57]
**Total (95% CI)**			**1018**			**1016**	**100.0%**	**-1.29 [-2.56, -0.01]**
Heterogeneity: Tau² = 1.54; Chi² = 18.07, df = 7 (P = 0.01); I² = 61%								
Test for overall effect: Z = 1.98 (P = 0.05)								

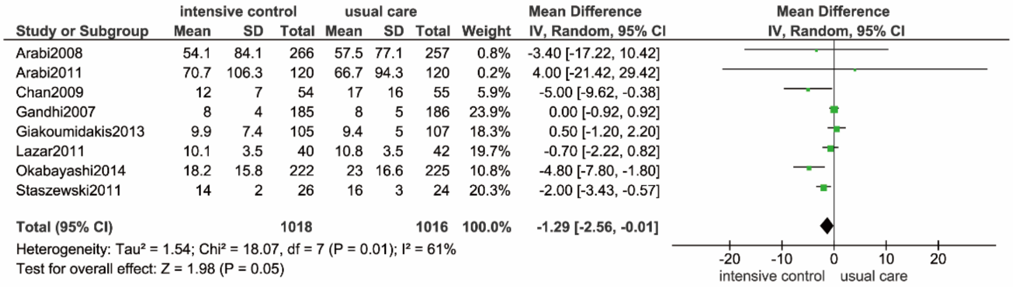

Supplementary Figure S12.

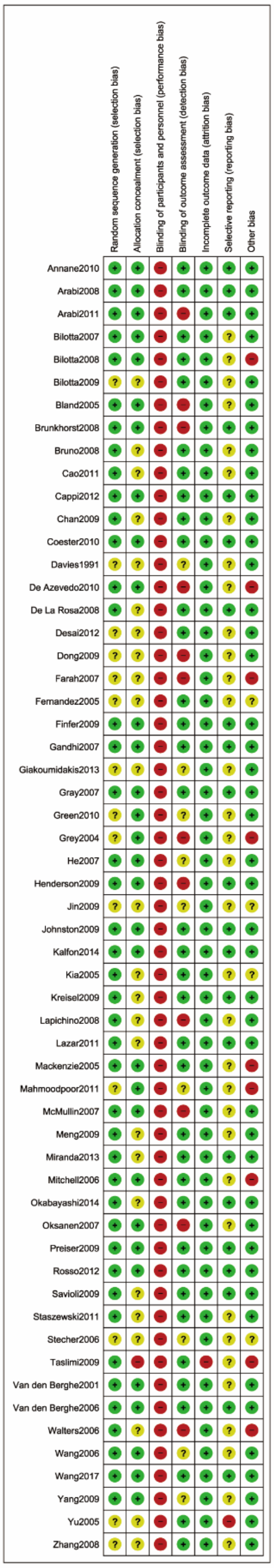

Supplementary Figure S13.

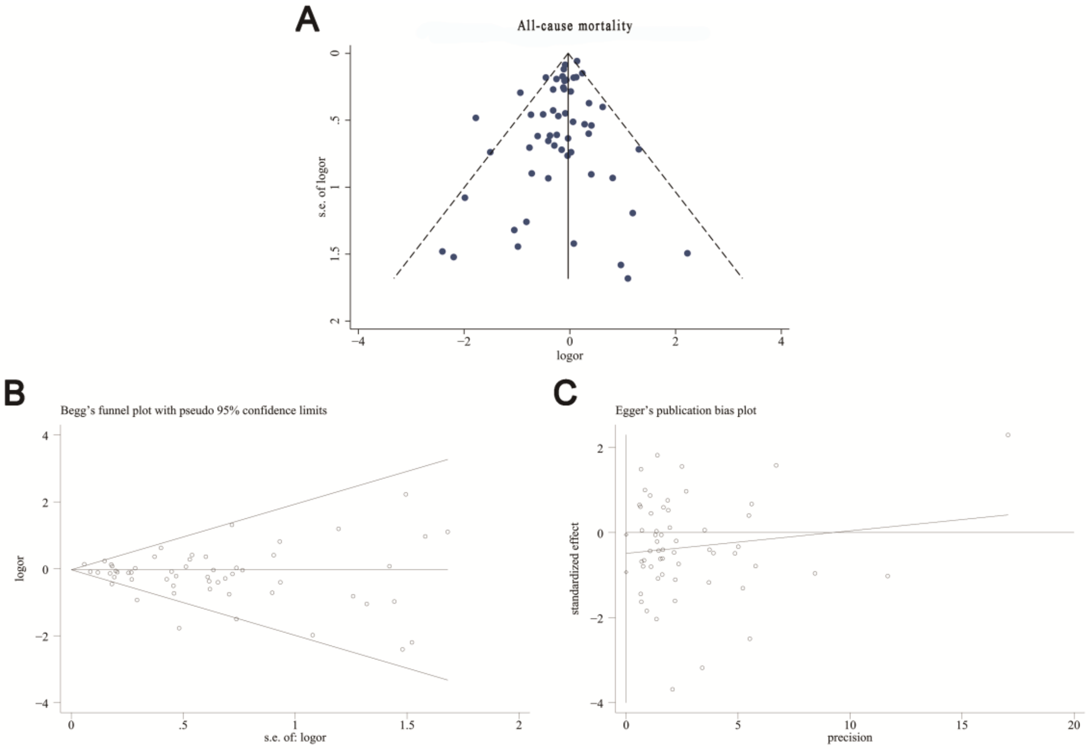

Supplementary Figure S14.

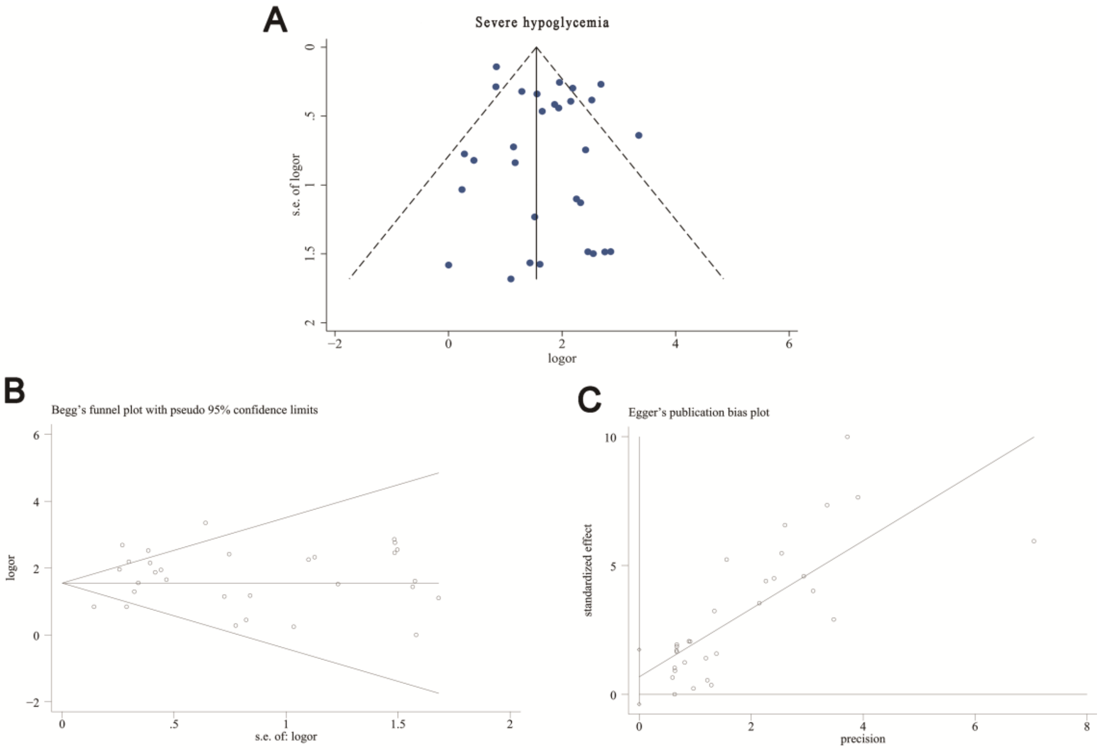

PRISMA (Preferred Reporting Items for Systematic Reviews and Meta-Analyses) Checklist

www.prisma-statement.org

You must report the page number in your manuscript where you consider each of the items listed in this checklist. If you have not included this information, either revise your manuscript accordingly before submitting or note N/A.

Section/Topic	Item No.	Checklist item	Reported on Page No.
**TITLE**			
Title	1	Identify the report as a systematic review, meta-analysis, or both.	1
**ABSTRACT**			
Structured summary	2	Provide a structured summary including, as applicable: background; objectives; data sources; study eligibility criteria, participants, and interventions; study appraisal and synthesis methods; results; limitations; conclusions and implications of key findings; systematic review registration number.	2-3
**INTRODUCTION**			
Rationale	3	Describe the rationale for the review in the context of what is already known.	4-6
Objectives	4	Provide an explicit statement of questions being addressed with reference to participants, interventions, comparisons, outcomes, and study design (PICOS).	4-6
**METHODS**			
Protocol and registration	5	Indicate if a review protocol exists, if and where it can be accessed (e.g., Web address), and, if available, provide registration information including registration number.	N/A
Eligibility criteria	6	Specify study characteristics (e.g., PICOS, length of follow-up) and report characteristics (e.g., years considered, language, publication status) used as criteria for eligibility, giving rationale.	6
Information sources	7	Describe all information sources (e.g., databases with dates of coverage, contact with study authors to identify additional studies) in the search and date last searched.	6
Search	8	Present full electronic search strategy for at least one database, including any limits used, such that it could be repeated.	6
Study selection	9	State the process for selecting studies (i.e., screening, eligibility, included in systematic review, and, if applicable, included in the meta-analysis).	7
Data collection process	10	Describe method of data extraction from reports (e.g., piloted forms, independently, in duplicate) and any processes for obtaining and confirming data from investigators.	7-8
Data items	11	List and define all variables for which data were sought (e.g., PICOS, funding sources) and any assumptions and simplifications made.	8-9
Risk of bias in individual studies	12	Describe methods used for assessing risk of bias of individual studies (including specification of whether this was done at the study or outcome level), and how this information is to be used in any data synthesis.	8

Section/Topic	Item No.	Checklist item	Reported on Page No.
Summary measures	13	State the principal summary measures (e.g., risk ratio, difference in means).	10-11
Synthesis of results	14	Describe the methods of handling data and combining results of studies, if done, including measures of consistency (e.g., I^2) for each meta-analysis.	10-11
Risk of bias across studies	15	Specify any assessment of risk of bias that may affect the cumulative evidence (e.g., publication bias, selective reporting within studies).	11
Additional analyses	16	Describe methods of additional analyses (e.g., sensitivity or subgroup analyses, meta-regression), if done, indicating which were pre-specified.	9-11
**RESULTS**			
Study selection	17	Give numbers of studies screened, assessed for eligibility, and included in the review, with reasons for exclusions at each stage, ideally with a flow diagram.	11-12
Study characteristics	18	For each study, present characteristics for which data were extracted (e.g., study size, PICOS, follow-up period) and provide the citations.	12
Risk of bias within studies	19	Present data on risk of bias of each study and, if available, any outcome level assessment (see item 12).	18
Results of individual studies	20	For all outcomes considered (benefits or harms), present, for each study: (a) simple summary data for each intervention group (b) effect estimates and confidence intervals, ideally with a forest plot.	12-16
Synthesis of results	21	Present results of each meta-analysis done, including confidence intervals and measures of consistency.	13-16
Risk of bias across studies	22	Present results of any assessment of risk of bias across studies (see Item 15).	18
Additional analysis	23	Give results of additional analyses, if done (e.g., sensitivity or subgroup analyses, meta-regression [see Item 16]).	15-17
**DISCUSSION**			
Summary of evidence	24	Summarize the main findings including the strength of evidence for each main outcome; consider their relevance to key groups (e.g., healthcare providers, users, and policy makers).	19-24
Limitations	25	Discuss limitations at study and outcome level (e.g., risk of bias), and at review-level (e.g., incomplete retrieval of identified research, reporting bias).	24-25
Conclusions	26	Provide a general interpretation of the results in the context of other evidence, and implications for future research.	25-26
**FUNDING**			

Section/Topic	Item No.	Checklist item	Reported on Page No.
Funding	27	Describe sources of funding for the systematic review and other support (e.g., supply of data); role of funders for the systematic review.	26

From: Moher D, Liberati A, Tetzlaff J, Altman DG, The PRISMA Group (2009). Preferred Reporting Items for Systematic Reviews and Meta-Analyses: The PRISMA Statement. PLoS Med 6(6): e1000097. doi:10.1371/journal.pmed1000097

Once you have completed this checklist, please save a copy and upload it as part of your submission. Please DO NOT include this checklist as part of the main manuscript document. It must be uploaded as a separate file.
